# Supplementary material for: Self-Assessment Instruments for Supporting Family Caregivers: An Integrative Review
Source: Healthcare (Basel). 2024 May 14;12(10):1016. doi: 10.3390/healthcare12101016 (PMC11120749; doi:10.3390/healthcare12101016)
Supplement: Supplementary file 1 [file healthcare-12-01016-s001.zip › healthcare-2935572-supplementary_1.pdf]

healthcare

MDPI

Supplementary 1

Database searches

PubMed Database Search.

**Instrument**	(Patient Reported Outcome Measures[MeSH Terms] OR Surveys[MeSH Terms] AND Questionnaires[MeSH Terms] OR self report[MeSH Terms])
	AND
**Purpose of the instrument**	(needs assessment[MeSH Terms] OR social support[MeSH Terms] OR cost of illness[MeSH Terms] OR Stress Disorders, Traumatic[MeSH Terms] OR Stress Disorders, Post-Traumatic[MeSH Terms] OR Burnout, Psychological[MeSH Terms])
	AND
**Family Caregivers**	(caregivers[MeSH Terms] OR family[MeSH Terms] OR friends[MeSH Terms])
	AND
**Setting**	(community health services[MeSH Terms] OR home care services[MeSH Terms])

Filters: Language: English, German, French, Italian.

Google Scholar (English):

#1 app "family caregiver" stress burden strain, Filter: Publication date from 2015

#2 "Needs assessment" "family caregiver" "Patient reported outcome measures",

Filter: Publication date from 2015

#3 "Needs assessment" "family caregiver" survey and questionnaires

Filter: Publication date from 2015

#4 "Needs assessment" "family caregiver" "self report", Filter: Publication date from 2015

#5 "Needs assessment" caregivers "self report", Filter: Publication date from 2015

#6 "Needs assessment" caregivers "patient reported outcome measures"

Filter: Publication date from 2015

#7 "Needs assessment" caregivers survey and questionnaires, Filter: Publication date from 2015

#8 "Needs assessment" caregivers survey and questionnaires

Filter: Publication date from 2010 to 2014

#9 "Needs assessment" caregivers survey and questionnaires

Filter: Publication date from 2000 to 2009

#10 "Social support" Caregiver "Home care services", Filter: Publication date from 2015

#11 "Social support" Caregiver "Home care services", Filter: Publication date from 2010 to 2014

#12 Stress "stress disorder" caregiver "self report", Filter: Publication date from 2015

#13 Stress caregiver "self report" survey and questionnaire, Filter: Publication date from 2015

#14 Stress "stress disorder" family friends measures, Filter: Publication date from 2015

#15 Stress "family caregiver" friends family measures, Filter: Publication date from 2015

#16 Stress "family caregiver" "patient reported outcome", Filter: Publication date from 2015

#17 Exhaustion "family caregiver" "self-report", Filter: Publication date from 2015

#18 Exhaustion caregiver "survey and questionnaire", Filter: Publication date from 2015

#19 Exhaustion "family caregiver" "patient reported outcome", Filter: Publication date from 2015

#20 Exhaustion burden strain "family caregiver" measures, Filter: Publication date from 2015

#21 "social support" caregiver questionnaire "self report", Filter: Publication date from 2015

#22 "social support" "family caregiver" measures, Filter: Publication date from 2015

#23 "social support" family friends caregiver "patient reported outcome measures"
Filter: Publication date from 2015

#24 "social support" family friends caregiver questionnaire, Filter: Publication date from 2015

#25 Reference search from identified hits

Google Scholar (German):

#1 Fragebogen Belastung "pflegende Angehörige", Filter: Publication date from 2015

#2 Fragebogen Belastung "pflegende Angehörige", Filter: Publication date from 2010 to 2014

#3 Instrument Selbsteinschätzung Angehörige Familienmitglieder

#4 Selbstcheck Angehörige

#5 Selbsttest "pflegende Angehörige" Unterstützung und Support

#6 Fragebogen Selbsteinschätzung von Angehörigen Freunde Familie

#7 Reference search from identified hits

Google IT:

#1 autovalutazione - questionario - pressione psicologica - familiari assistenti - Cura e assistenza a casa

#2 applicazione che aiuta a identificare lo stress psicologico dei familiari assistenti

#3 questionario che aiuta a identificare lo stress psicologico dei familiari assistenti

#4 Reference search from identified hits

Google FR:

#1 une application qui permet d'identifier le stress psychologique des aidants familiaux,

Filter: Publication date from 2019

#2 auto-évaluation – auto-évaluation – questionnaire - stress psychologique - proches aidants - à domicile

#3 auto-évaluation – auto-évaluation – questionnaire - stress psychologique - proches aidants - à domicile, Filter: Publication date from 2017

#4 Reference search from identified hits

Google CH:

#1 Fragebogen Selbsteinschätzung physische und psychische Erschöpfung "pflegende Angehörige

#2 Fragebogen zur Selbsteinschätzung von Angehörigen zum Bedarf

Filter: Publication date from 01.01.2017

#3 Selbsttest "pflegende Angehörige" Unterstützung Support

Filter: Publication date from 01.01.2017

#4 Selbstcheck Instrument pflegende Angehörige, Filter: Publication date from 01.01.2017

#5 Selbstcheck Fragebogen Familie Freunde "häusliche Pflege"

#6 Selbstcheck Familie "pflegende Angehörige"

#7 Selbsttest "pflegende Angehörige" Belastung, Filter: Publication date from 01.01.2017

#8 Selbstcheck Erschöpfung "pflegende Angehörige" Familie Freunde

#9 Fragebogen Eigeneinschätzung Angehörige Stabilisierung der häuslichen Pflege

#10 Instrument zur Selbsteinschätzung von Angehörigen in Pflegesituationen

#11 Instrument zum Selbsttest von Familienmitgliedern zur Edukation Beratung Prävention

Filter: Publication date from 01.01.2017

#12 Fragebogen Selbstfürsorge Unterstützung "pflegende Angehörige"

#13 Fragebogen Selbstmanagement Familienmitglieder

#14 Selbsteinschätzung Selbstfürsorge pflegende Angehörige Freunde Nachbarn Familie

#15 Fragebogen "pflegende Angehörige" Belastung

#16 Reference search from identified hits

Google AT:

#1 Selbsteinschätzung Belastung "pflegende Angehörige", Filter: Publication date from 01.01.2017

#2 Fragebogen Selbsteinschätzung physische und psychische Erschöpfung "pflegende Angehörige"

#3 Selbsteinschätzung Selbstfürsorge pflegende Angehörige Freunde Nachbarn Familie

#4 Fragebogen Selbstfürsorge Unterstützung "pflegende Angehörige"

#5 Fragebogen Selbstmanagement Familienmitglieder

#6 Selbstcheck Instrument pflegende angehörige

#7 Selbstcheck Fragebogen Familie Freunde "häusliche Pflege"

#8 Selbsttest "pflegende Angehörige" Belastung

#9 Selbsttest "pflegende Angehörige" Unterstützung Support

Filter: Publication date from 01.01.2017

#10 Fragebogen zur Selbsteinschätzung von Angehörigen zum Bedarf

#11 Instrument Selbsttest Edukation Beratung Prävention Betreuende Angehörige

Filter: Publication date from 01.01.2017

#12 Selbstcheck Erschöpfung "pflegende Angehörige" Familie Freunde

#13 Selbstcheck Erschöpfung "pflegende Angehörige"

#14 Fragebogen Eigeneinschätzung Stabilisierung der häuslichen Pflege

#15 Instrument zur Selbsteinschätzung von Angehörigen

#16 Reference search from identified hits

Google DE:

#1 Fragebogen Selbsteinschätzung physische und psychische Erschöpfung "pflegende Angehörige"

#2 Instrument zur Selbstfürsorge "pflegende Angehörige"

Filter: Publication date from 01.01.2017

#3 Fragebogen Selbstfürsorge Unterstützung "pflegende Angehörige"

Filter: Publication date from 01.01.2017

#4 Selbsteinschätzung Selbstfürsorge pflegende Angehörige Freunde Nachbarn Familie

#5 Fragebogen Selbstmanagement Familienmitglieder

#6 Selbsteinschätzung Bedarfserhebung betreuende Angehörige

#7 Selbstcheck Instrument pflegende Angehörige, Filter: Publication date from 01.01.2017

#8 Selbstcheck Instrument pflegende Angehörige, Filter: Publication date from 2010-2016

#9 Fragebogen Selbsteinschätzung "pflegende Angehörige"

#10 Instrument Selbsttest Edukation Beratung Prävention Betreuende Angehörige
Filter: Publication date from 01.01.2017

#11 Fragebogen zum selbst ausfüllen für Angehörige "häusliche Pflege"
Filter: Publication date from 01.01.2017

#12 Selbsteinschätzung Fragebogen Pflege Zuhause Belastung angehörige

#13 Selbsteinschätzung Belastung "pflegende Angehörige"

#14 Fragebogen zur Selbsteinschätzung von Angehörigen zum Bedarf

#15 Selbsttest "pflegende Angehörige" Unterstützung Support
Filter: Publication date from 01.01.2017

#16 Reference search from identified hits

Apps and further hand search:

#1 Zeitschrift für Gerontologie und Geriatrie, Pflege, Diagnostica, Testzentrale

#2 Google App Store with the search terms: pflegende Angehörige, Pflegebedürftigkeit, Pflege Zuhause, Angehörige Belastung, caregiver, care at home, burden

#3 Apple App Store with the search terms: Pflegebedürftigkeit, Pflege Zuhause, Angehörige Belastung, caregiver, care at home, burden

#4 Microsoft App Store nach Stichworten: Pflegebedürftigkeit, Pflege Zuhause, Angehörige Belastung, caregiver, care at home, burden

#5 Screening of results sections and reference lists of identified literature papers and systematic literature reviews.

#6 Contacting selected institutions, organizations and experts
