# Supplementary material for: Self-Assessment Instruments for Supporting Family Caregivers: An Integrative Review
Source: Healthcare (Basel). 2024 May 14;12(10):1016. doi: 10.3390/healthcare12101016 (PMC11120749; doi:10.3390/healthcare12101016)
Supplement: Supplementary file 1 [file healthcare-12-01016-s001.zip › healthcare-2935572-supplementary_2.pdf]

Supplementary File S2: References for included instruments *(n=45)*

This supplement lists the publications in which the instruments were used for all included instruments.

References

1 German assessment to survey resources of and risks to elderly family caregivers (Assessment zur Erfassung von Ressourcen und Risiken älterer pflegender Angehöriger – ARR) – Budnick, Kummer, Blüher & Dräger [30]:

[30] Budnick, A.; Kummer, K.; Blüher, S.; Dräger, D. Pflegende Angehörige und Gesundheitsförderung. Pilotstudie zur Validität eines deutschsprachigen Assessments zur Erfassung von Ressourcen und Risiken älterer pflegender Angehöriger (ARR). *Z. Für Gerontol. Und Geriatr.* **2012**, *45*, 201–211. https://doi.org/10.1007/s00391-011-0278-y.

2 Brief Assessment Scale for Caregiver (BASC) – Glajchen, Kornblith, Homel, Fraidin, Mauskop & Portenoy [31]:

[31] Glajchen, M.; Kornblith, A.; Homel, P.; Fraidin, L.; Mauskop, A.; Portenoy, R.K. Development of a brief assessment scale for caregivers of the medically ill. *J. Pain Symptom Manag.* **2005**, *29*, 245–254. https://doi.org/10.1016/j.jpainsymman.2004.06.017.

[99] Hudson, P.; Quinn, K.; Kristjanson, L.; Thomas, T.; Braithwaite, M.; Fisher, J.; Cockayne, M. Evaluation of a psycho-educational group programme for family caregivers in home-based palliative care. *Palliative Medicine* **2008**, *22*, 270–280, doi:10.1177/0269216307088187.

3 Burden Assessment Scale (BAS) – Reinhard, Gubman, Horwitz & Minsky [32]:

[31] Glajchen, M.; Kornblith, A.; Homel, P.; Fraidin, L.; Mauskop, A.; Portenoy, R.K. Development of a brief assessment scale for caregivers of the medically ill. *J. Pain Symptom Manag.* **2005**, *29*, 245–254. https://doi.org/10.1016/j.jpainsymman.2004.06.017.

[32] Reinhard, S.C.; Gubman, G.D.; Horwitz, A.V.; Minsky, S. Burden assessment scale for families of the seriously mentally ill. *Eval. Program Plan.* **1994**, *17*, 261–269. https://doi.org/10.1016/0149-7189(94)90004-3.

[100] Ivarsson, A.-B.; Sidenvall, B.; Carlsson, M. The factor structure of the Burden Assessment Scale and the perceived burden of caregivers for individuals with severe mental disorders. *Scandinavian Journal of Caring Sciences* **2004**, *18*, 396–401, doi:10.1111/j.1471-6712.2004.00298.x.

[101] Secinti, E.; Yavuz, H.M.; Selcuk, B. Feelings of burden among family caregivers of people with spinal cord injury in Turkey. *Spinal Cord* **2017**, *55*, 782–787, doi:10.1038/sc.2017.6.

4 Burden Assessment Schedule (BAS) – Thara, Padmavati, Kumar & Srinivasan [33]:

[102] Chadda, R.K.; Singh, T.B.; Ganguly, K.K. Caregiver burden and coping: a prospective study of relationship between burden and coping in caregivers of patients with schizophrenia and bipolar affective disorder. *Social Psychiatry and Psychiatric Epidemiology* **2007**, *42*, 923–930, doi:10.1007/s00127-007-0242-8.

[103] Karim, N.; Ali, A.; Deuri, S.P. A comparative study of care burden and social support among caregivers of persons with schizophrenia and epilepsy. *Open Journal of Psychiatry & Allied Sciences* **2015**, *6*, 132, doi:10.5958/2394-2061.2015.00011.7.

5 Burden Scale for Family Caregivers (BSFC) – Grässel & Leutbecher [34]:

[104] Auzinger, M. Physische und psychische Belastungen pflegender Angehöriger im Bezirk Braunau am Inn; UMIT, Hall in Tirol, 2009. Retrieved from https://www.oegkv.at/fileadmin/user_upload/Publikationen/Diplomarbeiten/AUZINGER_Maria_DA2009_Physische_und_psychische_Belastungen_pflegender_Angehoeriger_im_Bezirk_Braunau_am_Inn.pdf

[105] Brogaard, T.; Neergaard, M.A.; Guldin, M.-B.; Sokolowski, I.; Vedsted, P. Translation, adaptation and data quality of a Danish version of the Burden Scale for Family Caregivers. *Scandinavian Journal of Caring Sciences* **2013**, *27*, 1018–1026, doi:10.1111/j.1471-6712.2012.01092.x.

[106] Graessel E.; Berth, H.; Lichte, T.; Grau, H. Subjective caregiver burden: validity of the 10-item short version of the Burden Scale for Family Caregivers BSFC-s. *BMC Geriatrics* **2014**, *14*, 23, doi:10.1186/1471-2318-14-23.

[107] Gräsel, E. Somatic symptoms and caregiving strain among family caregivers of older patients with progressive nursing needs. *Archives of Gerontology and Geriatrics* **1995**, *21*, 253–266, doi:10.1016/0167-4943(95)00660-d.

[108] Gräsel E.; Chiu, T.; Oliver, R. *Development and Validation of the Burden Scale for Family Caregivers (BSFC);* Comprehensive Rehabilitation and Mental Health Services: Toronto, 2003.

[109] Gräßel, E. *"Burden Scale for Family Caregivers" in 20 Sprachen*, o.J (02.05.2020). Available online: www.caregiver-burden.eu.

[110] Grässel, E. Häusliche Pflege dementiell und nicht dementiell Erkrankter. Teil II: Gesundheit und Belastung der Pflegenden. *Zeitschrift für Gerontologie und Geriatrie* **1998**, *31*, 57–62, doi:10.1007/s003910050019.

[111] Gräßel, E. *HPS - Häusliche Pflege-Skala;* Hogrefe: Bern, 2001.

[112] Gräßel, E.; Behrndt, E.-M. Belastungen und Entlastungsangebote für pflegende Angehörige. *Pflege-Report 2016: Die Pflegenden im Fokus*; pp 169–187.

[113] Grau, H.; Graessel, E.; Berth, H. The subjective burden of informal caregivers of persons with dementia: extended validation of the German language version of the Burden Scale for Family Caregivers (BSFC). *Aging & Mental Health* **2015**, *19*, 159–168, doi:10.1080/13607863.2014.920296.

[114] Hecht, M.J.; Graesel, E.; Tigges, S.; Hillemacher, T.; Winterholler, M.; Hilz, M.-J.; Heuss, D.; Neundörfer, B. Burden of care in amyotrophic lateral sclerosis. *Palliative Medicine* **2003**, *17*, 327–333, doi:10.1191/0269216303pm754oa.

[115] Krug, K.; Miksch, A.; Peters-Klimm, F.; Engeser, P.; Szecsenyi, J. Correlation between patient quality of life in palliative care and burden of their family caregivers: a prospective observational cohort study. *BMC Palliative Care* **2016**, *15*, 2006, doi:10.1186/s12904-016-0082-y.

[116] Lamparter, C. *Die Belastung pflegender Angehöriger: Entwicklung eines Instrumentes zur Belastungseinschätzung pflegender Angehöriger von Demenzerkrankten*, 2019 (03.05.2020). Available online: https://dg-pflegewissenschaft.de/wp-content/uploads/2019/12/Session-7-Pr%C3%A4sentation-Carmen-Lamparter.pdf.

[117] Pendergrass, A.; Malnis, C.; Graf, U.; Engel, S.; Graessel, E. Screening for caregivers at risk: Extended validation of the short version of the Burden Scale for Family Caregivers (BSFC-s) with a valid classification system for caregivers caring for an older person at home. *BMC Health Services Research* **2018**, *18*, 76, doi:10.1186/s12913-018-3047-4.

6 Cancer Caregiving Tasks, Consequences and Needs Questionnaire (CaTCoN) – Lund, Ross & Groenvold [35]:

[35] Lund, L.; Ross, L.; Groenvold, M. The initial development of the 'Cancer Caregiving Tasks, Consequences and Needs Questionnaire' (CaTCoN). *Acta Oncol.* **2012**, *51*, 1009–1019. https://doi.org/10.3109/0284186x.2012.681697.

[118] Lund, L.; Ross, L.; Petersen, M.A.; Groenvold, M. The validity and reliability of the 'Cancer Caregiving Tasks, Consequences and Needs Questionnaire' (CaTCoN). *Acta Oncologica* **2014**, *53*, 966–974, doi:10.3109/0284186X.2014.888496.

[119] Zavagli, V.; Raccichini, M.; Ercolani, G.; Franchini, L.; Varani, S.; Pannuti, R. Care for Carers: an Investigation on Family Caregivers' Needs, Tasks, and Experiences. *Translational Medicine* **2019**, *19*, 54–59.

6 Caregiver Burden Inventory (CBI) – Novak & Guest [36]:

[49] Barello, S.; Castiglioni, C.; Bonanomi, A.; Graffigna, G. The Caregiving Health Engagement Scale (CHE-s): Development and initial validation of a new questionnaire for measuring family caregiver engagement in healthcare. *BMC Public Health* **2019**, *19*, 1562. https://doi.org/10.1186/s12889-019-7743-8.

[79] Chiatti, C.; Masera, F.; Rimland, J.M.; Cherubini, A.; Scarpino, O.; Spazzafumo, L.; Lattanzio, F. The UP-TECH project, an intervention to support caregivers of Alzheimer's disease patients in Italy: Study protocol for a randomized controlled trial. *Trials* **2013**, *14*, 155. https://doi.org/10.1186/1745-6215-14-155.

[120] Akpınar, B.; Küçükgüçlü, O.; Yener, G. Effects of gender on burden among caregivers of Alzheimer's patients. *Journal of Nursing Scholarship* **2011**, *43*, 248–254, doi:10.1111/j.1547-5069.2011.01402.x.

[121] Caserta, M.S.; Lund, D.A.; Wright, S.D. Exploring the Caregiver Burden Inventory (CBI): further evidence for a multidimensional view of burden. *International Journal of Aging & Human Development* **1996**, *43*, 21–34, doi:10.2190/2dkf-292p-a53w-w0a8.

[122] Chien, W.T.; Lee, I.Y.M. Randomized controlled trial of a dementia care programme for families of home-resided older people with dementia. *Journal of Advanced Nursing* **2011**, *67*, 774–787, doi:10.1111/j.1365-2648.2010.05537.x.

[123] Chiò, A.; Vignola, A.; Mastro, E.; Dei Giudici, A.; Iazzolino, B.; Calvo, A.; Moglia, C.; Montuschi, A. Neurobehavioral symptoms in ALS are negatively related to caregivers' burden and quality of life. *European Journal of Neurology* **2010**, *17*, 1298–1303, doi:10.1111/j.1468-1331.2010.03016.x.

[124] D'Onofrio, G.; Sancarlo, D.; Addante, F.; Ciccone, F.; Cascavilla, L.; Paris, F.; Picoco, M.; Nuzzaci, C.; Elia, A.C.; Greco, A.; et al. Caregiver burden characterization in patients with Alzheimer's disease or vascular dementia. *International Journal of Geriatric Psychiatry* **2015**, *30*, 891–899, doi:10.1002/gps.4232.

[125] Evans, R.; Catapano, M.A.; Brooks, D.; Goldstein, R.S.; Avendano, M. Family caregiver perspectives on caring for ventilator-assisted individuals at home. *Canadian Respiratory Journal* **2012**, *19*, 373–379, doi:10.1155/2012/452898.

[126] Fianco, A.; Sartori, R.D.G.; Negri, L.; Lorini, S.; Valle, G.; Delle Fave, A. The relationship between burden and well-being among caregivers of Italian people diagnosed with severe neuromotor and cognitive disorders. *Research in Developmental Disabilities* **2015**, *39*, 43–54, doi:10.1016/j.ridd.2015.01.006.

[127] Gauthier, A.; Vignola, A.; Calvo, A.; Cavallo, E.; Moglia, C.; Sellitti, L.; Mutani, R.; Chiò, A. A longitudinal study on quality of life and depression in ALS patient-caregiver couples. *Neurology* **2007**, *68*, 923–926, doi:10.1212/01.wnl.0000257093.53430.a8.

[128] Greco, A.; Pancani, L.; Sala, M.; Annoni, A.M.; Steca, P.; Paturzo, M.; D'Agostino, F.; Alvaro, R.; Vellone, E. Psychometric characteristics of the caregiver burden inventory in caregivers of adults with heart failure. *European Journal of Cardiovascular Nursing* **2017**, *16*, 502–510, doi:10.1177/1474515117693890.

[129] Iavarone, A.; Ziello, A.R.; Pastore, F.; Fasanaro, A.M.; Poderico, C. Caregiver burden and coping strategies in caregivers of patients with Alzheimer's disease. *Neuropsychiatric Disease and Treatment* **2014**, *10*, 1407–1413, doi:10.2147/ndt.S58063.

[130] Lai, C.; Cipriani, M.; Renzi, A.; Luciani, M.; Lombardo, L.; Aceto, P. The Effects of the Perception of Being Recognized by Patients With Alzheimer Disease on a Caregiver's Burden and Psychophysical Health. *The American journal of hospice & palliative care* **2018**, *35*, 1188–1194, doi:10.1177/1049909118766316.
[131] Marvardi, M.; Mattioli, P.; Spazzafumo, L.; Mastriforti, R.; Rinaldi, P.; Polidori, M.C.; Cherubini, A.; Quartesan, R.; Bartorelli, L.; Bonaiuto, S.; et al. The Caregiver Burden Inventory in evaluating the burden of caregivers of elderly demented patients: results from a multicenter study. *Aging Clinical and Experimental Research* **2005**, *17*, 46–53, doi:10.1007/BF03337720.
[132] McCleery, A.; Addington, J.; Addington, D. Family assessment in early psychosis. *Psychiatry Research* **2007**, *152*, 95–102, doi:10.1016/j.psychres.2006.07.002.
[133] Merluzzi, T.V.; Philip, E.J.; Vachon, D.O.; Heitzmann, C.A. Assessment of self-efficacy for caregiving: The critical role of self-care in caregiver stress and burden. *Palliative & Supportive Care* **2011**, *9*, 15–24, doi:10.1017/S1478951510000507.
[134] Reckrey, J.M.; DeCherrie, L.V.; Kelley, A.S.; Ornstein, K. Health Care Utilization Among Homebound Elders. *Journal of Aging and Health* **2013**, *25*, 1036–1049, doi:10.1177/0898264313497509.
[135] Rosa, E.; Ambrogio, M.; Binetti, G.; Zanetti, O. 'Immigrant paid caregivers' and primary caregivers' burden. *International Journal of Geriatric Psychiatry* **2004**, *19*, 1103–1104, doi:10.1002/gps.1176.
[136] Sinforiani, E.; Pasotti, C.; Chiapella, L.; Malinverni, P.; Zucchella, C. Differences between physician and caregiver evaluations in Alzheimer's disease. *Functional Neurology* **2010**, *25*, 205–209.
[137] Tramonti, F.; Barsanti, I.; Bongioanni, P.; Bogliolo, C.; Rossi, B. A permanent emergency: A longitudinal study on families coping with amyotrophic lateral sclerosis. *Families, Systems, & Health* **2014**, *32*, 271–279, doi:10.1037/fsh0000032.

7 Caregiver Burden Scale (CBS) – Elmstahl, Malmberg & Annerstedt [37]:

[37] Elmståhl, S.; Malmberg, B.; Annerstedt, L. Caregiver's burden of patients 3 years after stroke assessed by a novel caregiver burden scale. *Arch. Phys. Med. Rehabil.* **1996**, *77*, 177–182. https://doi.org/10.1016/s0003-9993(96)90164-1.
[138] Andren, S.; Elmstahl, S. Family caregivers' subjective experiences of satisfaction in dementia care: aspects of burden, subjective health and sense of coherence. *Scandinavian Journal of Caring Sciences* **2005**, *19*, 157–168, doi:10.1111/j.1471-6712.2005.00328.x.
[139] Annerstedt L.; Elmståhl S.; Ingvad, B.; Samuelsson, S.M. Family caregiving in dementia--an analysis of the caregiver's burden and the "breaking-point" when home care becomes inadequate. *Scandinavian Journal of Public Health* **2000**, *28*, 23–31, doi:10.1177/140349480002800106.
[140] Belasco, A.; Barbosa, D.; Bettencourt, A.R.; Diccini, S.; Sesso, R. Quality of life of family caregivers of elderly patients on hemodialysis and peritoneal dialysis. *American Journal of Kidney Diseases* **2006**, *48*, 955–963, doi:10.1053/j.ajkd.2006.08.017.
[141] Cil Akinci, A.; Pinar, R. Validity and reliability of Turkish Caregiver Burden Scale among family caregivers of haemodialysis patients. *Journal of Clinical Nursing* 2014, *23*, 352–360, doi:10.1111/j.1365-2702.2012.04235.x.
[142] Elmstahl, S.; Ingvad, B.; Annerstedt, L. Family caregiving in dementia: prediction of caregiver burden 12 months after relocation to group-living care. *International Psychogeriatrics* **1998**, *10*, 127–146, doi:10.1017/s1041610298005249.
[143] Mashayekhi, F.; Jozdani, R.H.; Chamak, M.N.; Mehni, S. Caregiver Burden and Social Support in Mothers with β-Thalassemia Children. *Global Journal of Health Science* **2016**, *8*, 206, doi:10.5539/gjhs.v8n12p206.
[144] Olai, L.; Borgquist, L.; Svärdsudd, K. Life situations and the care burden for stroke patients and their informal caregivers in a prospective cohort study. *Upsala Journal of Medical Sciences* **2015**, *120*, 290–298, doi:10.3109/03009734.2015.1049388.

8 Caregiver Burden Screen (CBS) – Rankin, Haut, Keefover & Franzen [38]:

[145] Redinbaugh, E.M.; Baum, A.; Tarbell, S.; Arnold, R. End-of-life caregiving: what helps family caregivers cope? *Journal of Palliative Medicine* **2003**, *6*, 901–909, doi:10.1089/109662103322654785.

10 Caregiver Needs Screen (CNS) – Boele, Terhorst, Prince, Donovan, Weimer & Sherwood et al. [39]:

[39] Boele, F.W.; Terhorst, L.; Prince, J.; Donovan, H.S.; Weimer, J.; Sherwood, P.R.; Lieberman, F.S.; Drappatz, J. Psychometric Evaluation of the Caregiver Needs Screen in Neuro-Oncology Family Caregivers. *J. Nurs. Meas.* **2019**, *27*, 162–176. https://doi.org/10.1891/1061-3749.27.2.162.

11 Caregiver Reaction Assessment (CRA) – Given, Given, Stommel, Collins, King & Franklin [40]:

[39] Boele, F.W.; Terhorst, L.; Prince, J.; Donovan, H.S.; Weimer, J.; Sherwood, P.R.; Lieberman, F.S.; Drappatz, J. Psychometric Evaluation of the Caregiver Needs Screen in Neuro-Oncology Family Caregivers. *J. Nurs. Meas.* **2019**, *27*, 162–176. https://doi.org/10.1891/1061-3749.27.2.162.
[40] Given, C.W.; Given, B.; Stommel, M.; Collins, C.; King, S.; Franklin, S. The caregiver reaction assessment (CRA) for caregivers to persons with chronic physical and mental impairments. *Res. Nurs. Health* **1992**, *15*, 271–283. https://doi.org/10.1002/nur.4770150406.
[73] Gaugler, J.E.; Anderson, K.A.; Leach, M.S.W.C.R.; Smith, C.D.; Schmitt, F.A.; Mendiondo, M. The emotional ramifications of unmet need in dementia caregiving. *Am. J. Alzheimers Dis. Other Dement.* **2004**, *19*, 369–380. https://doi.org/10.1177/153331750401900605.
[146] Aggar, C.; Ronaldson, S.; Cameron, I.D. Reactions to caregiving during an intervention targeting frailty in community living older people. *BMC Geriatrics* **2012**, *12*, 66, doi:10.1186/1471-2318-12-66.

[147] Bachner, Y.G.; O'Rourke, N.; Carmel, S. Psychometric properties of a modified version of the Caregiver Reaction Assessment Scale measuring caregiving and post-caregiving reactions of caregivers of cancer patients. *Journal of Palliative Care* **2007**, *23*, 80–86.
[148] Dudgeon, D.J.; Knott, C.; Eichholz, M.; Gerlach, J.L.; Chapman, C.; Viola, R.; van Dijk, J.; Preston, S.; Batchelor, D.; Bartfay, E. Palliative Care Integration Project (PCIP) quality improvement strategy evaluation. *Journal of Pain and Symptom Management* **2008**, *35*, 573–582, doi:10.1016/j.jpainsymman.2007.07.013.
[149] Frias, C.M. de; Tuokko, H.; Rosenberg, T. Caregiver physical and mental health predicts reactions to caregiving. *Aging & Mental Health* **2005**, *9*, 331–336, doi:10.1080/13607860500089674.
[150] Gaugler, J.E.; Hanna, N.; Linder, J.; Given, C.; Tolbert, V.; Kataria, R.; Regine, W.F. Cancer caregiving and subjective stress: a multi-site, multi-dimensional analysis. *Psycho-Oncology* **2005**, *14*, 771–785, doi:10.1002/pon.916.
[151] Grater, J.J. The impact of health care provider communication on self -efficacy and caregiver burden in older spousal oncology caregivers - ProQuest; Pittsburgh, University of, 2005.
[152] Grov, E.K.; Eklund, M.L. Reactions of primary caregivers of frail older people and people with cancer in the palliative phase living at home. *Journal of Advanced Nursing* **2008**, *63*, 576–585, doi:10.1111/j.1365-2648.2008.04736.x.
[153] Grov, E.K.; Fosså, S.D.; Sørebø, O.; Dahl, A.A. Primary caregivers of cancer patients in the palliative phase: a path analysis of variables influencing their burden. *Social Science & Medicine* **2006**, *63*, 2429–2439, doi:10.1016/j.socscimed.2006.06.008.
[154] Grov, E.K.; Fosså, S.D.; Tønnessen, A.; Dahl, A.A. The caregiver reaction assessment: psychometrics, and temporal stability in primary caregivers of Norwegian cancer patients in late palliative phase. *Psycho-Oncology* **2006**, *15*, 517–527, doi:10.1002/pon.987.
[155] Hudson, P.L.; Thomas, K.; Trauer, T.; Remedios, C.; Clarke, D. Psychological and social profile of family caregivers on commencement of palliative care. *Journal of Pain and Symptom Management* **2011**, *41*, 522–534, doi:10.1016/j.jpainsymman.2010.05.006.
[156] Ishii, Y.; Miyashita, M.; Sato, K.; Ozawa, T. Family's Difficulty Scale in End-of-Life Home Care: A New Measure of the Family's Difficulties in Caring for Patients with Cancer at the End of Life at Home from Bereaved Family's Perspective. *Journal of Palliative Medicine* **2012**, *15*, 210–215, doi:10.1089/jpm.2011.0248.
[157] Jacobi, C.E.; van den Berg, B.; Boshuizen, H.C.; Rupp, I.; Dinant, H.J.; van den Bos, G.A.M. Dimension-specific burden of caregiving among partners of rheumatoid arthritis patients. *Rheumatology (Oxford, England)* **2003**, *42*, 1226–1233, doi:10.1093/rheumatology/keg366.
[158] Kristanti, M.S.; Vernooij-Dassen, M.; Utarini, A.; Effendy, C.; Engels, Y. Measuring the Burden on Family Caregivers of People With Cancer. *Cancer Nursing* **2020**, 1, doi:10.1097/NCC.0000000000000733.
[159] Lambert, S.D.; Yoon, H.; Ellis, K.R.; Northouse, L. Measuring appraisal during advanced cancer: Psychometric testing of the appraisal of caregiving scale. *Patient Education and Counseling* **2015**, *98*, 633–639, doi:10.1016/j.pec.2015.01.009.
[160] Lethin, C.; Renom-Guiteras, A.; Zwakhalen, S.; Soto-Martin, M.; Saks, K.; Zabalegui, A.; Challis, D.J.; Nilsson, C.; Karlsson, S. Psychological well-being over time among informal caregivers caring for persons with dementia living at home. *Aging & Mental Health* **2017**, *21*, 1138–1146, doi:10.1080/13607863.2016.1211621.
[161] Luttik, M.L.; Jaarsma, T.; Veeger, N.; Tijssen, J.; Sanderman, R.; van Veldhuisen, D.J. Caregiver burden in partners of Heart Failure patients; limited influence of disease severity. *European Journal of Heart Failure* **2007**, *9*, 695–701, doi:10.1016/j.ejheart.2007.01.006.
[162] Misawa, T.; Miyashita, M.; Kawa, M.; Abe, K.; Abe, M.; Nakayama, Y.; Given, C.W. Validity and Reliability of the Japanese Version of the Caregiver Reaction Assessment Scale (CRA-J) for Community-Dwelling Cancer Patients. *American Journal of Hospice and Palliative Medicine®* **2009**, *26*, 334–340, doi:10.1177/1049909109338480.
[163] Nijboer, C.; Triemstra, M.; Tempelaar, R.; Mulder, M.; Sanderman, R.; van den Bos, G.A. Patterns of Caregiver Experiences Among Partners of Cancer Patients. *The Gerontologist* **2000**, *40*, 738–746, doi:10.1093/geront/40.6.738.
[164] Nijboer, C.; Triemstra, M.; Tempelaar, R.; Sanderman, R.; van den Bos, G.A.M. Determinants of caregiving experiences and mental health of partners of cancer patients. *Cancer* **1999**, *86*, 577–588, doi:10.1002/(SICI)1097-0142(19990815)86:4<577:AID-CNCR6>3.0.CO;2-S.
[165] Nijboer, C.; Triemstra, M.; Tempelaar, R.; Sanderman, R.; van den Bos, G.A. Measuring both negative and positive reactions to giving care to cancer patients: psychometric qualities of the Caregiver Reaction Assessment (CRA). *Social Science & Medicine* **1999**, *48*, 1259–1269, doi:10.1016/S0277-9536(98)00426-2.
[166] Persson, C., Wennman-Larsen, A., Sundin, K., & Gustavsson, P. Assessing informal caregivers' experiences: a qualitative and psychometric evaluation of the Caregiver Reaction Assessment Scale. *European Journal of Cancer Care* **2008**, *17*, 189–199, doi:10.1111/j.1365-2354.2007.00833.x.
[167] Saunders, M.M. Factors Associated with Caregiver Burden in Heart Failure Family Caregivers. *Western Journal of Nursing Research* **2008**, *30*, 943–959, doi:10.1177/0193945908319990.
[168] Sautter, J.M.; Tulsky, J.A.; Johnson, K.S.; Olsen, M.K.; Burton-Chase, A.M.; Hoff Lindquist, J.; Zimmerman, S.; Steinhauser, K.E. Caregiver Experience During Advanced Chronic Illness and Last Year of Life. *Journal of the American Geriatrics Society* **2014**, *62*, 1082–1090, doi:10.1111/jgs.12841.
[169] Soares, A.J.; Ferreira, G.; Graça Pereira, M. Depression, distress, burden and social support in caregivers of active versus abstinent addicts. *Addiction Research & Theory* **2016**, *24*, 483–489, doi:10.3109/16066359.2016.1173681.

[170] Stein, R.A.; Sharpe, L.; Bell, M.L.; Boyle, F.M.; Dunn, S.M.; Clarke, S.J. Randomized Controlled Trial of a Structured Intervention to Facilitate End-of-Life Decision Making in Patients With Advanced Cancer. *Journal of Clinical Oncology* **2013**, *31*, 3403–3410, doi:10.1200/JCO.2011.40.8872.

[171] van Exel, N.J.A.; Scholte op Reimer, W. J. M.; Brouwer, W.B.F.; van den Berg, B.; Koopmanschap, M.A.; van den Bos, G.A. Instruments for assessing the burden of informal caregiving for stroke patients in clinical practice: a comparison of CSI, CRA, SCQ and self-rated burden. *Clinical Rehabilitation* **2004**, *18*, 203–214, doi:10.1191/0269215504cr723oa.

12 Caregiver Risk Screen (CRS) – Guberman, Keefe, Fancey, Nahmiash & Barylak [41]:

[172] Huyck, M.H.; Ayalon, L.; Yoder, J. Using mixed methods to evaluate the use of a caregiver strain measure to assess outcomes of a caregiver support program for caregivers of older adults. *International Journal of Geriatric Psychiatry* **2007**, *22*, 160–165, doi:10.1002/gps.1707.

13 Caregiver Self-Assessment Questionnaire (CSAQ) – American Medical Association [42]:

[42] American Medical Association. AMA Homepage. Available online: https://www.ama-assn.org/ (accessed on 12 May 2020).

14 Caregiver Strain Index (CSI) – Robinson [43]:

[43] Robinson, B.C. Validation of a Caregiver Strain Index. *J. Gerontol.* **1983**, *38*, 344–348. https://doi.org/10.1093/geronj/38.3.344.

[173] Adriaansen, J.J.E.; van Leeuwen, C.M.C.; Visser-Meily, J.M.A.; van den Bos, G.A.M.; Post, M.W.M. Course of social support and relationships between social support and life satisfaction in spouses of patients with stroke in the chronic phase. *Patient Education and Counseling* **2011**, *85*, e48-52, doi:10.1016/j.pec.2010.12.011.

[174] Akosile, C.O.; Banjo, T.O.; Okoye, E.C.; Ibikunle, P.O.; Odole, A.C. Informal caregiving burden and perceived social support in an acute stroke care facility. *Health and Quality of Life Outcomes* **2018**, *16*, 57, doi:10.1186/s12955-018-0885-z.

[175] Bonner, M.J.; Hardy, K.K.; Guill, A.B.; McLaughlin, C.; Schweitzer, H.; Carter, K. Development and validation of the parent experience of child illness. *Journal of Pediatric Psychology* **2006**, *31*, 310–321, doi:10.1093/jpepsy/jsj034.

[176] Brannan, A.M.; Heflinger, C.A.; Bickman, L. The Caregiver Strain Questionnaire. *Journal of Emotional and Behavioral Disorders* **1997**, *5*, 212–222, doi:10.1177/106342669700500404.

[177] Brazil, K.; Thabane, L.; Foster, G.; Bédard, M. Gender differences among Canadian spousal caregivers at the end of life. *Health & Social Care in the Community* **2009**, *17*, 159–166, doi:10.1111/j.1365-2524.2008.00813.x.

[178] Chen, M.-L.; Hu, L.-C. The generalizability of Caregiver Strain Index in family caregivers of cancer patients. *International Journal of Nursing Studies* **2002**, *39*, 823–829, doi:10.1016/s0020-7489(02)00021-4.

[179] Comans, T.A.; Currin, M.L.; Brauer, S.G.; Haines, T.P. Factors associated with quality of life and caregiver strain amongst frail older adults referred to a community rehabilitation service: implications for service delivery. *Disability and Rehabilitation* **2011**, *33*, 1215–1221, doi:10.3109/09638288.2010.525288.

[180] Creemers, H.; Morée, S. de; Veldink, J.H.; Nollet, F.; van den Berg, L.H.; Beelen, A. Factors related to caregiver strain in ALS: a longitudinal study. *Journal of Neurology, Neurosurgery, and Psychiatry* **2016**, *87*, 775–781, doi:10.1136/jnnp-2015-311651.

[181] del-Pino-Casado, R.; Millán-Cobo, M.D.; Palomino-Moral, P.A.; Frías-Osuna, A. Cultural correlates of burden in primary caregivers of older relatives: a cross-sectional study. *Journal of Nursing Scholarship* **2014**, *46*, 176–186, doi:10.1111/jnu.12070.

[182] Diwan, S.; Hougham, G.W.; Sachs, G.A. Strain experienced by caregivers of dementia patients receiving palliative care: findings from the Palliative Excellence in Alzheimer Care Efforts (PEACE) Program. *Journal of Palliative Medicine* **2004**, *7*, 797–807, doi:10.1089/jpm.2004.7.797.

[183] Heru, A.M.; Ryan, C.E. Family functioning in the caregivers of patients with dementia: one-year follow-up. *Bulletin of the Menninger Clinic* **2006**, *70*, 222–231, doi:10.1521/bumc.2006.70.3.222.

[184] Hwang, S.S.; Chang, V.T.; Alejandro, Y.; Osenenko, P.; Davis, C.; Cogswell, J.; Srinivas, S.; Kasimis, B. Caregiver unmet needs, burden, and satisfaction in symptomatic advanced cancer patients at a Veterans Affairs (VA) medical center. *Palliative & Supportive Care* **2003**, *1*, 319–329, doi:10.1017/s1478951503030475.

[185] Jenkinson, C.; Fitzpatrick, R.; Swash, M.; Peto, V.; and the ALS-HPS Steering Group. The ALS Health Profile Study: quality of life of amyotrophic lateral sclerosis patients and carers in Europe. *Journal of Neurology* **2000**, *247*, 835–840, doi:10.1007/s004150070069.

[186] Keefe, F.J.; Ahles, T.A.; Sutton, L.; Dalton, J.; Baucom, D.; Pope, M.S.; Knowles, V.; McKinstry, E.; Furstenberg, C.; Syrjala, K.; et al. Partner-Guided Cancer Pain Management at the End of Life: A Preliminary Study. *Journal of Pain and Symptom Management* **2005**, *29*, 263–272, doi:10.1016/j.jpainsymman.2004.06.014.

[187] Kim, M.-D.; Hong, S.-C.; Lee, C.-I.; Kim, S.-Y.; Kang, I.-O.; Lee, S.-Y. Caregiver burden among caregivers of Koreans with dementia. *Gerontology* **2009**, *55*, 106–113, doi:10.1159/000176300.

[188] Kochaki Nejad, Z.; Mohajjel Aghdam, A.; Hassankhani, H.; Sanaat, Z. The Effects of a Patient-Caregiver Education and Follow-Up Program on the Breast Cancer Caregiver Strain Index. *Iranian Red Crescent Medical Journal* **2016**, *18*, e21627-e21627, doi:10.5812/ircmj.21627.

[189] Kruithof, W.J.; Post, M.W.; van Mierlo, M.L.; van den Bos, G.A.; Man-van Ginkel, J.M. de; Visser-Meily, J.M. Caregiver burden and emotional problems in partners of stroke patients at two months and one year post-stroke: Determinants and prediction. *Patient Education and Counseling* **2016**, *99*, 1632–1640, doi:10.1016/j.pec.2016.04.007.

[190] McPherson, K.M.; Pentland, B.; McNaughton, H.K. Brain injury - the perceived health of carers. *Disability and Rehabilitation* **2000**, *22*, 683–689, doi:10.1080/096382800445489.
[191] Miaskowski, C.; Zimmer, E.F.; Barrett, K.M.; Dibble, S.L.; Wallhagen, M. Differences in patients' and family caregivers' perceptions of the pain experience influence patient and caregiver outcomes. *Pain* **1997**, *72*, 217–226, doi:10.1016/S0304-3959(97)00037-7.
[192] Payne, S.; Smith, P.; Dean, S. Identifying the concerns of informal carers in palliative care. *Palliative Medicine* **1999**, *13*, 37–44, doi:10.1191/026921699673763725.
[193] Rettke, H.; Geschwindner, H.M. Long-term outcomes of stroke rehabilitation: patients and informal caregivers. *Pflege* **2014**, *27*, 131–133, doi:10.1024/1012-5302/a000353.
[194] Schumacher, K.L.; Dodd, M.J.; Paul, S.M. The stress process in family caregivers of persons receiving chemotherapy. *Research in Nursing & Health* **1993**, *16*, 395–404, doi:10.1002/nur.4770160603.

15 Caregiver Tasks Inventory – Clark & Rakowski [44] :

[195] Chen, H.-C.; Chen, M.-L.; Lotus Shyu, Y.; Tang, W.-R. Development and testing of a scale to measure caregiving load in caregivers of cancer patients in Taiwan, the care task scale-cancer. *Cancer Nursing* **2007**, *30*, 223–231, doi:10.1097/01.Ncc.0000270701.49543.64.

16 Caregiver's Burden Scale in End-of-Life Care (CBS-EOLC) – Dumont, Fillion, Gagnon & Bernier [45]:

[196] Brazil, K.; Kaasalainen, S.; Williams, A.; Rodriguez, C. *Comparing the experiences of rural and urban family caregivers of the terminally ill*, 2013.

17 Caregiving Appraisal Scale (CAS) – Lawton, Kleban, Moss, Rovine & Glicksman [46, 47]:

[46] Lawton, M.P.; Kleban, M.H.; Moss, M.; Rovine, M.; Glicksman, A. Measuring Caregiving Appraisal. *J. Gerontol.* **1989**, *44*, P61–P71. https://doi.org/10.1093/geronj/44.3.P61.
[57] Cooper, B.; Kinsella, G.J.; Picton, C. Development and initial validation of a family appraisal of caregiving questionnaire for palliative care. *Psycho-Oncol.* **2006**, *15*, 613–622. https://doi.org/10.1002/pon.1001.
[121] Caserta, M.S.; Lund, D.A.; Wright, S.D. Exploring the Caregiver Burden Inventory (CBI): further evidence for a multidimensional view of burden. *International Journal of Aging & Human Development* **1996**, *43*, 21–34, doi:10.2190/2dkf-292p-a53w-w0a8.
[187] Kim, M.-D.; Hong, S.-C.; Lee, C.-I.; Kim, S.-Y.; Kang, I.-O.; Lee, S.-Y. Caregiver burden among caregivers of Koreans with dementia. *Gerontology* **2009**, *55*, 106–113, doi:10.1159/000176300.
[197] Albert, S.M.; Im, A.; Brenner, L.; Smith, M.; Waxman, R. Effect of a social work liaison program on family caregivers to people with brain injury. *The Journal of Head Trauma Rehabilitation* **2002**, *17*, 175–189, doi:10.1097/00001199-200204000-00007.
[198] Edmonds, P.; Hart, S.; Wei, G.; Vivat, B.; Burman, R.; Silber, E.; Higginson, I.J. Palliative care for people severely affected by multiple sclerosis: evaluation of a novel palliative care service. *Multiple sclerosis* **2010**, *16*, 627–636, doi:10.1177/1352458510364632.
[199] Farran, C.J.; Miller, B.H.; Kaufman, J.E.; Donner, E.; Fogg, L. Finding meaning through caregiving: development of an instrument for family caregivers of persons with Alzheimer's disease. *Journal of Clinical Psychology* **1999**, *55*, 1107–1125, doi:10.1002/(sici)1097-4679(199909)55:9<1107:aid-jclp8>3.0.co;2-v.
[200] Findeis, A.; Larson, J.L.; Gallo, A.; Shekleton, M. Caring for individuals using home ventilators: an appraisal by family caregivers. *Rehabilitation Nursing* **1994**, *19*, 6–11, doi:10.1002/j.2048-7940.1994.tb01295.x.
[201] Stolley, J.M.; Reed, D.; Buckwalter, K.C. Caregiving appraisal and interventions based on the progressively lowered stress threshold model. *American Journal of Alzheimer's Disease and other Dementias* **2002**, *17*, 110–120, doi:10.1177/153331750201700211.

18 Caregiving Hassles Scale (CHS) – Kinney & Stephens [48]:

[48] Kinney, J.M.; Stephens, M.A.P. Caregiving Hassles Scale: Assessing the Daily Hassles of Caring for a Family Member With Dementia1. *Gerontologist* **1989**, *29*, 328–332. https://doi.org/10.1093/geront/29.3.328.

19 Caregiving Health Engagement Scale (CHE-s) – Barello, Castiglioni, Bonanomi & Graffigna [49]:

[49] Barello, S.; Castiglioni, C.; Bonanomi, A.; Graffigna, G. The Caregiving Health Engagement Scale (CHE-s): Development and initial validation of a new questionnaire for measuring family caregiver engagement in healthcare. *BMC Public Health* **2019**, *19*, 1562. https://doi.org/10.1186/s12889-019-7743-8.

19 Caregiving Stress Appraisal Scale (CSA) – Abe [50]:

[50] Abe, K. Reconsidering the Caregiving Stress Appraisal scale: Validation and examination of its association with items used for assessing long-term care insurance in Japan. *Arch. Gerontol. Geriatr.* **2007**, *44*, 287–297. https://doi.org/10.1016/j.archger.2006.06.001.
[202] Affinito, J. Recognizing burden in unpaid caregivers of patients with end stage renal disease. Dissertation; William Paterson University of New Jersey, 2016.

20 Carer Experience Scale (CES) – Al-Janabi. Coast, & Flynn [51]:

[203] Hoefman, R.; Al-Janabi, H.; McCaffrey, N.; Currow, D.; Ratcliffe, J. Measuring caregiver outcomes in palliative care: a construct validation study of two instruments for use in economic evaluations. *Quality of Life Research* **2015**, *24*, 1255–1273, doi:10.1007/s11136-014-0848-8.

21 Carer's Assessment of Difficulties Index (CADI) – Nolan & Grant [53]:

[204] Charlesworth, G.M.; Tzimoula, X.M.; Newman, S.P. Carers Assessment of Difficulties Index (CADI): psychometric properties for use with carers of people with dementia. *Aging & Mental Health* **2007**, *11*, 218–225, doi:10.1080/13607860600844523.

[205] Coudin, G.; Mollard, J. Difficulties, coping strategies and satisfactions in family caregivers of people with Alzheimer's disease. *Geriatrie et psychologie neuropsychiatrie du vieillissement* **2011**, *9*, 363–378, doi:10.1684/pnv.2011.0286.

[206] Levesque, J.V.; Maybery, D.J. The Parental Cancer Questionnaire: scale structure, reliability, and validity. *Supportive Care in Cancer* **2014**, *22*, 23–32, doi:10.1007/s00520-013-1935-z.

[207] Loke, A.Y.; Liu, C.-F.F.; Szeto, Y. The Difficulties Faced by Informal Caregivers of Patients With Terminal Cancer in Hong Kong and the Available Social Support. *Cancer Nursing* **2003**, *26*, 276–283, doi:10.1097/00002820-200308000-00004.

[208] Mafullul, Y.M. Burden of informal carers of mentally infirm eldery in Lancashire. *East African Medical Journal* **2002**, *79*, 291–298, doi:10.4314/eamj.v79i6.8848.

[209] Mollard, Judith. (2011). Nolan Methode: CADI, CAMI, CASI. Retrieved from https://pdfs.semanticscholar.org/519b/740a43df671258cd6575cfe236f453c22636.pdf **2011**.

[210] Sequeira, C. Difficulties, coping strategies, satisfaction and burden in informal Portuguese caregivers. *Journal of Clinical Nursing* **2013**, *22*, 491–500, doi:10.1111/jocn.12108.

22 Carer's Checklist – Hodgson, Higginson & Jefferys [54]:

[54] Hodgson, C.; Higginson, I.; Jefferys, P. *Carers Checklist*; The Mental Health Foundation: London, UK, 1998. Retrieved from https://www.mentalhealth.org.uk/sites/default/files/carers_checklist.pdf

24 Cope-Index (COPE) – McKee, Philp, Lamura, Prouskas. Oberg, Krevers et al. & COPE Partnership [55]:

[55] McKee, K.J.; Philp, I.; Lamura, G.; Prouskas, C.; Oberg, B.; Krevers, B.; Spazzafumo, L.; Bień, B.; Parker, C.; Nolan, M.R.; et al. The COPE index--a first stage assessment of negative impact, positive value and quality of support of caregiving in informal carers of older people. *Aging Ment. Health* **2003**, *7*, 39–52. https://doi.org/10.1080/1360786021000006956.

[78] Chiatti, C.; Di Rosa, M.; Melchiorre, M.G.; Manzoli, L.; Rimland, J.M.; Lamura, G. Migrant care workers as protective factor against caregiver burden: Results from a longitudinal analysis of the EUROFAMCARE study in Italy. *Aging Ment. Health* **2013**, *17*, 609–614. https://doi.org/10.1080/13607863.2013.765830.

[211] Balducci, C.; Mnich, E.; McKee, K.J.; Lamura G.; Beckmann, A.; Krevers, B.; Wojszel, Z.B.; Nolan, M.; Prouskas, C.; Bien, B.; et al. Negative impact and positive value in caregiving: validation of the COPE index in a six-country sample of carers. *The Gerontologist* **2008**, *48*, 276–286, doi:10.1093/geront/48.3.276.

[212] Deutsche Gesellschaft für Allgemeinmedizin und Familienmedizin e.V. *Pflegende Angehörige von Erwachsenen: S3-Leitlinie* (03.05.2020). Available online: https://www.awmf.org/uploads/tx_szleitlinien/053-006l_S3_Pflegende-Angehoerige-von-Erwachsenen_2019-03.pdf.

[213] Kofahl, C. Determinanten der Belastung, Entlastung und Lebensqualität betreuender Angehöriger älterer Menschen. Dissertation; Hamburg, Universität.

[214] Roud, H., Keeling, S.; Sainsbury, R. Using the COPE assessment tool with informal carers of people with dementia in New Zealand. *New Zealand Medical Journal* **2006**, *119*, 1237.

25 Coping Inventory (CI) – Barusch [56]:

[187] Kim, M.-D.; Hong, S.-C.; Lee, C.-I.; Kim, S.-Y.; Kang, I.-O.; Lee, S.-Y. Caregiver burden among caregivers of Koreans with dementia. *Gerontology* **2009**, *55*, 106–113, doi:10.1159/000176300.

26 Family Appraisal of Caregiving Questionnaire for Palliative Care (FACQ-PC) – Cooper, Kinsella & Picton [57]:

[57] Cooper, B.; Kinsella, G.J.; Picton, C. Development and initial validation of a family appraisal of caregiving questionnaire for palliative care. *Psycho-Oncol.* **2006**, *15*, 613–622. https://doi.org/10.1002/pon.1001.

[215] Aoun, S.M.; Grande, G.; Howting, D.; Deas, K.; Toye, C.; Troeung, L.; Stajduhar, K.; Ewing, G. The impact of the carer support needs assessment tool (CSNAT) in community palliative care using a stepped wedge cluster trial. *PloS one* **2015**, *10*, e0123012, doi:10.1371/journal.pone.0123012.

[216] Ewing, G.; Brundle, C.; Payne S.; Grande, G. The Carer Support Needs Assessment Tool (CSNAT) for use in palliative and end-of-life care at home: a validation study. *Journal of Pain and Symptom Management* **2013**, *46*, 395–405, doi:10.1016/j.jpainsymman.2012.09.008.

[217] Janssen, D.J.; Spruit, M.A.; Wouters, E.F.; Schols, J.M. Family Caregiving in Advanced Chronic Organ Failure. *Journal of the American Medical Directors Association* **2012**, *13*, 394–399, doi:10.1016/j.jamda.2011.04.017.

27 Family Burden Scale (FBS) – Madianos, Economou, Dafni, Koukia, Palli & Rogakou [58]:

[58] Madianos, M.; Economou, M.; Dafni, O.; Koukia, E.; Palli, A.; Rogakou, E. Family disruption, economic hardship and psychological distress in schizophrenia: Can they be measured? *Eur. Psychiatry J. Assoc. Eur. Psychiatr.* **2004**, *19*, 408–414. https://doi.org/10.1016/j.eurpsy.2004.06.028.

28 Family Caregiver Distress Assessment Tool – Home Instead [59]:

[59] Home Instead. S.C. Family Caregiver Distress Assessment Tool. Available online: https://www.caregiverstress.com/stress-management/family-caregiver-stress/stress-assessment/ (accessed on 25 May 2020).

29 Heart Failure Caregiver Questionnaire (HF-CQ) – Strömberg, Bonner, Grant, Bennett, Chung, Jaarsma et al. [60]:

[60] Strömberg, A.; Bonner, N.; Grant, L.; Bennett, B.; Chung, M.L.; Jaarsma, T.; Luttik, M.L.; Lewis, E.F.; Calado, F.; Deschaseaux, C. Psychometric Validation of the Heart Failure Caregiver Questionnaire (HF-CQ®). *Patient* **2017**, *10*, 579–592. https://doi.org/10.1007/s40271-017-0228-x.

30 Hemophilia Caregiver Impact measure (HCI) – Schwartz, Powell & Eldar-Lissai [61]:

[61] Schwartz, C.E.; Powell, V.E.; Eldar-Lissai, A. Measuring hemophilia caregiver burden: Validation of the Hemophilia Caregiver Impact measure. *Qual. Life Res.* **2017**, *26*, 2551–2562. https://doi.org/10.1007/s11136-017-1572-y.

31 Impact of Event Scale (-Revised) (IES-R) – Weiss & Marmar [62]:

[175] Bonner, M.J.; Hardy, K.K.; Guill, A.B.; McLaughlin, C.; Schweitzer, H.; Carter, K. Development and validation of the parent experience of child illness. *Journal of Pediatric Psychology* **2006**, *31*, 310–321, doi:10.1093/jpepsy/jsj034.

[218] Kazak, A.E.; McClure, K.S.; Alderfer, M.A.; Hwang, W.-T.; Crump, T.A.; Le, L.T.; Deatrick, J.; Simms, S.; Rourke, M.T. Cancer-Related Parental Beliefs: The Family Illness Beliefs Inventory (FIBI). *Journal of Pediatric Psychology* **2004**, *29*, 531–542, doi:10.1093/jpepsy/jsh055.

32 Impact on Family Scale (IOFS) – Stein & Riessman [63]:

[175] Bonner, M.J.; Hardy, K.K.; Guill, A.B.; McLaughlin, C.; Schweitzer, H.; Carter, K. Development and validation of the parent experience of child illness. *Journal of Pediatric Psychology* **2006**, *31*, 310–321, doi:10.1093/jpepsy/jsj034.

[176] Ravens-Sieberer, U., Morfeld, M., Stein, R. E., Jessop, D. J., Bullinger, M., & Thyen, U. (2001). Der Familien-Belastungs-Fragebogen (FaBel-Fragebogen) - Testung und Validierung der deutschen Version der "Impact on Family Scale" bei Familien mit behinderten Kindern. [The testing and validation of the German version of the impact on family scale in families with children with disabilities]. Psychotherapie, Psychosomatik, medizinische Psychologie, 51(9-10), 384–393. doi:10.1055/s-2001-16899 **2001**, doi:10.1055/s-2001-16899.

33 Life Situation among Spouses after a Stroke Event Questionnaire (LISS-Q)
Larson, Franzen-Dahlin, Billing, Murray & Wredling [64]:

[64] Larson, J.; Franzén-Dahlin, Å.; Billing, E.; Murray, V.; Wredling, R. Spouse's life situation after partner's stroke event: Psychometric testing of a questionnaire. *J. Adv. Nurs.* **2005**, *52*, 300–306. https://doi.org/10.1111/j.1365-2648.2005.03590.x.

33 Modified Caregiver Strain Index (MCSI) – Thornton & Travis [65]:

[65] Thornton, M.; Travis, S.S. Analysis of the reliability of the modified caregiver strain index. *J. Gerontol. Ser. B Psychol. Sci. Soc. Sci.* **2003**, *58*, S127–S132. https://doi.org/10.1093/geronb/58.2.s127.

[220] Jennings, L.A.; Reuben, D.B.; Evertson, L.C.; Serrano, K.S.; Ercoli, L.; Grill, J.; Chodosh, J.; Tan, Z.; Wenger, N.S. Unmet Needs of Caregivers of Individuals Referred to a Dementia Care Program. *Journal of the American Geriatrics Society* **2015**, *63*, 282–289, doi:10.1111/jgs.13251.

[221] Onega, L.L. Helping Those Who Help Others. *American Journal of Nursing* **2008**, *108*, 62–69, doi:10.1097/01.NAJ.0000334528.90459.9a.

[222] Pecorelli, N.; Knapp, J. Leben mit einem Parkinsonkranken – Eine Gratwanderung zwischen Überfürsorglichkeit und Überforderung : «Welche Faktoren gibt es, die Einfluss auf die Lebensqualität pflegender Angehörigen von Parkinson-PatientInnen haben?». Bachelorarbeit; ZHAW Zürcher Hochschule für Angewandte Wissenschaften, 2015.

[223] Ribeiro, O.; Brandão, D.; Oliveira, A.F.; Martín, I.; Teixeira, L.; Paúl, C. The Modified Caregiver Strain Index: Portuguese version. *Journal of Health Psychology* **2019**, *59*, 135910531988393, doi:10.1177/1359105319883933.

35 Montgomery Borgatta caregiver burden scale (MBCBS) – Montgomery & Borgotta [66, 67]:

[66] Montgomery, R.J.; Borgotta, E.F. The effects of alternative support strategies on family caregiving. *Gerontologist* **1989**, *29*, 457–464. https://doi.org/10.1093/geront/29.4.457.
[67] Montgomery, R.J.; Gonyea, J.G.; Hooyman, N.R. Caregiving and the Experience of Subjective and Objective Burden. *Fam. Relat.* **1985**, *34*, 19. https://doi.org/10.2307/583753.
[224] Ampalam, P.; Gunturu, S.; Padma, V. A Comparative Study of Caregivers Burden in Psychiatric Illness and Chronic Medical Illness. *Indian Journal of Psychiatry* **2012**, *54*, 239–243.
[225] Elsa, V. Informal Family Caregiver Burden in Elderly Assistance and Nursing Implications. *Annals of Nursing and Practice* **2015**, *2*, 1017.
[226] Fujinami, R.; Sun, V.; Zachariah, F.; Uman, G.; Grant, M.; Ferrell, B. Family caregivers' distress levels related to quality of life, burden, and preparedness. *Psycho-Oncology* **2015**, *24*, 54–62, doi:10.1002/pon.3562.
[227] Garand, L.; Dew, M.A.; Eazor, L.R.; Dekosky, S.T.; Reynolds, C.F.3. Caregiving burden and psychiatric morbidity in spouses of persons with mild cognitive impairment. *International Journal of Geriatric Psychiatry* **2005**, *20*, 512–522, doi:10.1002/gps.1318.
[228] Grant, M.; Sun, V.; Fujinami, R.; Sidhu, R.; Otis-Green, S.; Juarez, G.; Klein, L.; Ferrell, B. Family caregiver burden, skills preparedness, and quality of life in non-small cell lung cancer. *Oncology Nursing Forum* **2013**, *40*, 337–346, doi:10.1188/13.Onf.337-346.
[229] Gridelli, C.; Ferrara, C.; Guerriero, C.; Palazzo, S.; Grasso, G.; Pavese, I.; Satta, F.; Bajetta, E.; Cortinovis, D.; Barbieri, F.; et al. Informal caregiving burden in advanced non-small cell lung cancer: the HABIT study. *Journal of Thoracic Oncology* **2007**, *2*, 475–480, doi:10.1097/01.JTO.0000275342.47584.f3.
[230] Institut universitaire de gériatrie de Montréal. *Version canadienne-française du "Montgomery Borgatta Caregiver Burden"* (03.05.2020). Available online: http://www.criugm.qc.ca/images/stories/outils_cliniques/CRIUGM_54.pdf.
[231] O'Hara, R.E.; Hull, J.G.; Lyons, K.D.; Bakitas, M.; Hegel, M.T.; Li, Z.; Ahles, T.A. Impact on caregiver burden of a patient-focused palliative care intervention for patients with advanced cancer. *Palliative & Supportive Care* **2010**, *8*, 395–404, doi:10.1017/S1478951510000258.
[232] Savundranayagam, M.Y.; Hummert, M.L.; Montgomery, R.J.V. Investigating the Effects of Communication Problems on Caregiver Burden. *The Gerontologist* **2005**, *60*, S48-S55, doi:10.1093/geronb/60.1.S48.
[233] Stone, L.J.; Clements, J.A. The Effects of Nursing Home Placement on the Perceived Levels of Caregiver Burden. *Journal of Gerontological Social Work* **2009**, *52*, 193–214, doi:10.1080/01634370802609163.

36 Needs Assessment of Family Caregivers-Cancer (NAFC-C) – Kim, Kashy, Spillers & Evans [68]:

[68] Kim, Y.; Kashy, D.A.; Spillers, R.L.; Evans, T.V. Needs assessment of family caregivers of cancer survivors: Three cohorts comparison. *Psycho-Oncol.* **2010**, *19*, 573–582. https://doi.org/10.1002/pon.1597.
[234] Lefranc, A.; Pérol, D.; Plantier, M.; Chatelain, P.; Rohan-Chabot, H. de; Schell, M. Assessment of informal caregiver's needs by self-administered instruments: a literature review. *European Journal of Public Health* **2017**, *27*, 796–801, doi:10.1093/eurpub/ckx103.

37 Parent Caregiver Strain Questionnaire (PCSQ) – England & Roberts [69]:

[69] England, M.; Roberts, B.L. Theoretical and psychometric analysis of caregiver strain. *Res. Nurs. Health* **1996**, *19*, 499–510. https://doi.org/10.1002/(sici)1098-240x(199612)19:6<499::Aid-nur5>3.0.Co;2-j.

38 Pearlin Stress Process Model – Pearlin, Mullan, Semple & Skaff [16]:

[16] Pearlin, L.I.; Mullan, J.T.; Semple, S.J.; Skaff, M.M. Caregiving and the stress process: An overview of concepts and their measures. *Gerontologist* **1990**, *30*, 583–594. https://doi.org/10.1093/geront/30.5.583.

[39] Boele, F.W.; Terhorst, L.; Prince, J.; Donovan, H.S.; Weimer, J.; Sherwood, P.R.; Lieberman, F.S.; Drappatz, J. Psychometric Evaluation of the Caregiver Needs Screen in Neuro-Oncology Family Caregivers. *J. Nurs. Meas.* **2019**, *27*, 162–176. https://doi.org/10.1891/1061-3749.27.2.162.
[99] Hudson, P.; Quinn, K.; Kristjanson, L.; Thomas, T.; Braithwaite, M.; Fisher, J.; Cockayne, M. Evaluation of a psycho-educational group programme for family caregivers in home-based palliative care. *Palliative Medicine* **2008**, *22*, 270–280, doi:10.1177/0269216307088187.
[150] Gaugler, J.E.; Hanna, N.; Linder, J.; Given, C.; Tolbert, V.; Kataria, R.; Regine, W.F. Cancer caregiving and subjective stress: a multi-site, multi-dimensional analysis. *Psycho-Oncology* **2005**, *14*, 771–785, doi:10.1002/pon.916.
[155] Hudson, P.L.; Thomas, K.; Trauer, T.; Remedios, C.; Clarke, D. Psychological and social profile of family caregivers on commencement of palliative care. *Journal of Pain and Symptom Management* **2011**, *41*, 522–534, doi:10.1016/j.jpainsymman.2010.05.006.
[193] Rettke, H.; Geschwindner, H.M. Long-term outcomes of stroke rehabilitation: patients and informal caregivers. *Pflege* **2014**, *27*, 131–133, doi:10.1024/1012-5302/a000353.
[199] Farran, C.J.; Miller, B.H.; Kaufman, J.E.; Donner, E.; Fogg, L. Finding meaning through caregiving: development of an instrument for family caregivers of persons with Alzheimer's disease. *Journal of Clinical Psychology* **1999**, *55*, 1107–1125, doi:10.1002/(sici)1097-4679(199909)55:9<1107:aid-jclp8>3.0.co;2-v.

[235] Campbell, P.; Wright, J.; Oyebode, J.; Job, D.; Crome, P.; Bentham, P.; Jones, L.; Lendon, C. Determinants of burden in those who care for someone with dementia. *International Journal of Geriatric Psychiatry* **2008**, *23*, 1078–1085, doi:10.1002/gps.2071.
[236] Carter, P.A.; Acton, G.J. Personality and coping: predictors of depression and sleep problems among caregivers of individuals who have cancer. *Journal of Gerontological Nursing* **2006**, *32*, 45–53, doi:10.3928/0098-9134-20060201-11.
[237] Chumbler, N.R.; Grimm, J.W.; Cody, M.; Beck, C. Gender, kinship and caregiver burden: the case of community-dwelling memory impaired seniors. *International Journal of Geriatric Psychiatry* **2003**, *18*, 722–732, doi:10.1002/gps.912.
[238] Greenberger, H.; Litwin, H. Can burdened caregivers be effective facilitators of elder care-recipient health care? *Journal of Advanced Nursing* **2003**, *41*, 332–341, doi:10.1046/j.1365-2648.2003.02531.x.
[239] Halm, M.A.; Treat-Jacobson, D.; Lindquist, R.; Savik, K. Caregiver burden and outcomes of caregiving of spouses of patients who undergo coronary artery bypass graft surgery. *Heart & Lung* **2007**, *36*, 170–187, doi:10.1016/j.hrtlng.2006.08.003.
[240] Löckenhoff, C.E.; Duberstein, P.R.; Friedman, B.; Costa, P.T. Five-factor personality traits and subjective health among caregivers: The role of caregiver strain and self-efficacy. *Psychology and Aging* **2011**, *26*, 592–604, doi:10.1037/a0022209.
[241] Nijboer, C.; Tempelaar, R.; Sanderman, R.; Triemstra, M.; Spruijt, R.J.; van den Bos, G.A. Cancer and caregiving: the impact on the caregiver's health. *Psycho-Oncology* **1998**, *7*, 3–13, doi:10.1002/(SICI)1099-1611(199801/02)7:1<3:AID-PON320>3.0.CO;2-5.
[242] Raina, P. The Health and Well-Being of Caregivers of Children With Cerebral Palsy. *Pediatrics* **2005**, *115*, e626-e636, doi:10.1542/peds.2004-1689.
[243] Singh, M.; Cameron, J. Psychosocial aspects of caregiving to stroke patients. *Axone (Dartmouth, N.S.)* **2005**, *27*, 18–24.
[244] Skaff, M.M.; Pearlin, L.I. Caregiving: Role Engulfment and the Loss of Self. *The Gerontologist* **1992**, *32*, 656–664, doi:10.1093/geront/32.5.656.

39 Perceived Stress Scale (PSS) – Cohen, Kamarck & Mermelstein [70]:

[107] Gräsel, E. Somatic symptoms and caregiving strain among family caregivers of older patients with progressive nursing needs. *Archives of Gerontology and Geriatrics* **1995**, *21*, 253–266, doi:10.1016/0167-4943(95)00660-d.
[133] Merluzzi, T.V.; Philip, E.J.; Vachon, D.O.; Heitzmann, C.A. Assessment of self-efficacy for caregiving: The critical role of self-care in caregiver stress and burden. *Palliative & Supportive Care* **2011**, *9*, 15–24, doi:10.1017/S1478951510000507.
[245] Bobbitt, S.A.; Baugh, L.A.; Andrew, G.H.; Cook, J.L.; Green, C.R.; Pei, J.R.; Rasmussen, C.R. Caregiver needs and stress in caring for individuals with fetal alcohol spectrum disorder. *Research in Developmental Disabilities* **2016**, *55*, 100–113, doi:10.1016/j.ridd.2016.03.002.
[246] Gratao, A.C.M.; Vendrúscolo, T.R.P.; Talmelli, Luana Flávia da Silva; Figueiredo, L.C.; Santos, J.L.F.; Rodrigues, R.A.P. Sobrecarga e desconforto emocional em cuidadores de idosos. *Enfermagem* **2012**, *21*, 304–312.
[247] Hsiao, C.-Y. Family demands, social support and caregiver burden in Taiwanese family caregivers living with mental illness: the role of family caregiver gender. *Journal of Clinical Nursing* **2010**, *19*, 3494–3503, doi:10.1111/j.1365-2702.2010.03315.x.
[248] King, A.C.; Brassington, G. Enhancing physical and psychological functioning in older family caregivers: The role of regular physical activity1,2. *Annals of Behavioral Bedicine* **1997**, *19*, 91–100, doi:10.1007/bf02883325.
[249] Klein, E.M.; Brähler, E.; Dreier, M.; Reinecke, L.; Müller, K.W.; Schmutzer, G.; Wölfling, K.; Beutel, M.E. The German version of the Perceived Stress Scale - psychometric characteristics in a representative German community sample. *BMC Psychiatry* **2016**, *16*, 159, doi:10.1186/s12888-016-0875-9.
[250] Pinquart, M.; Sörensen, S. Spouses, adult children, and children-in-law as caregivers of older adults: A meta-analytic comparison. *Psychology and Aging* **2011**, *26*, 1–14, doi:10.1037/a0021863.
[251] Roberge, M. Évolution des stratégies d'adaptation chez les conjointes aidantes en soins palliatifs à domicile: étude de cas mutiple. Doktorarbeit; Université de Sherbrooke, 2018.
[252] Schwarz, K.A.; Dunphy, G. An Examination of Perceived Stress in Family Caregivers of Older Adults with Heart Failure. *Experimental Aging Research* **2003**, *29*, 221–235, doi:10.1080/03610730303717.

40 Sekentei scale for caregivers (SSC) – Asahara, Momose, Murashima, Okubo & Magilvy [71]:

[71] Asahara, K.; Momose, Y.; Murashima, S.; Okubo, N.; Magilvy, J.K. The relationship of social norms to use of services and caregiver burden in Japan. *J. Nurs. Scholarsh.* **2001**, *33*, 375–380. https://doi.org/10.1111/j.1547-5069.2001.00375.x.

40 Selbsttest für pflegende Angehörige – Seniorplace GmbH [72]:

[72] Seniorplace. Selbsttest für Pflegende Angehörige. Available online: https://www.seniorplace.de/selbsttest.php#tab1 (accessed on 31 May 2020).

42 Self-developed unmet need measure – Gaugler, Anderson, Leach, Smith, Schmitt & Mendiondo [73]:

[73] Gaugler, J.E.; Anderson, K.A.; Leach, M.S.W.C.R.; Smith, C.D.; Schmitt, F.A.; Mendiondo, M. The emotional ramifications of unmet need in dementia caregiving. *Am. J. Alzheimers Dis. Other Dement.* **2004**, *19*, 369–380. https://doi.org/10.1177/153331750401900605.

43 Sense of Competence questionnaire (SCQ) – Vernooij-Dassen, Persoon & Felling [74]:

[74] Vernooij-Dassen, M.J.; Felling, A.J.; Brummelkamp, E.; Dauzenberg, M.G.; van den Bos, G.A.; Grol, R. Assessment of caregiver's competence in dealing with the burden of caregiving for a dementia patient: A Short Sense of Competence Questionnaire (SSCQ) suitable for clinical practice. *J. Am. Geriatr. Soc.* **1999**, *47*, 256–257. https://doi.org/10.1111/j.1532-5415.1999.tb04588.x.

[253] Meeuwsen, E.J.; Melis, R.J.F.; van der Aa, G.C.H.M.; Goluke-Willemse, G.A.M.; Leest, B.J.M. de; van Raak, F.H.J.M.; Scholzel-Dorenbos, C.J.M.; Verheijen, D.C.M.; Verhey, F.R.J.; Visser, M.C.; et al. Effectiveness of dementia follow-up care by memory clinics or general practitioners: randomised controlled trial. *BMJ* **2012**, *344*, e3086-e3086, doi:10.1136/bmj.e3086.

[254] Pendergrass, A.; Beische, D.; Becker, C.; Hautzinger, M.; Pfeiffer, K. An abbreviated German version of the Sense of Competence Questionnaire among informal caregivers of relatives who had a stroke: development and validation. *European Journal of Ageing* **2015**, *12*, 203–213, doi:10.1007/s10433-015-0342-3.

[255] Reimer, W. J. M. Scholte op; Haan, R.J. de; Rijnders, P.T.; Limburg, M.; van den Bos, G.A.M. The Burden of Caregiving in Partners of Long-Term Stroke Survivors. *Stroke* **1998**, *29*, 1605–1611, doi:10.1161/01.STR.29.8.1605.

43 Social Support Rating Scale (SSRS) – Xiao [75]:

[256] Hu, X.; Peng, X.; Su, Y.; Huang, W. Caregiver burden among Chinese family caregivers of patients with lung cancer: A cross-sectional survey. *European Journal of Oncology Nursing* **2018**, *37*, 74–80, doi:10.1016/j.ejon.2018.11.003.

[257] Qiu, R.M.; Tao, Y.; Zhou, Y.; Zhi, Q.H.; Lin, H.C. The relationship between children's oral health-related behaviors and their caregiver's social support. *BMC Oral Health* **2016**, *16*, 86, doi:10.1186/s12903-016-0270-4.

44 Zarit Burden Interview (ZBI) – Zarit, Reever & Bach-Peterson [76]:

[38] Rankin, E.D.; Haut, M.W.; Keefover, R.W.; Franzen, M.D. The establishment of clinical cutoffs in measuring caregiver burden in dementia. *Gerontologist* **1994**, *34*, 828–832. https://doi.org/10.1093/geront/34.6.828.

[45] Dumont, S.; Fillion, L.; Gagnon, P.; Bernier, N. A new tool to assess family caregivers' burden during end-of-life care. *J. Palliat. Care* **2008**, *24*, 151–161.

[76] Zarit, S.H.; Reever, K.E.; Bach-Peterson, J. Relatives of the impaired elderly: Correlates of feelings of burden. *Gerontologist* **1980**, *20*, 649–655. https://doi.org/10.1093/geront/20.6.649.

[160] Lethin, C.; Renom-Guiteras, A.; Zwakhalen, S.; Soto-Martin, M.; Saks, K.; Zabalegui, A.; Challis, D.J.; Nilsson, C.; Karlsson, S. Psychological well-being over time among informal caregivers caring for persons with dementia living at home. *Aging & Mental Health* **2017**, *21*, 1138–1146, doi:10.1080/13607863.2016.1211621.

[187] Kim, M.-D.; Hong, S.-C.; Lee, C.-I.; Kim, S.-Y.; Kang, I.-O.; Lee, S.-Y. Caregiver burden among caregivers of Koreans with dementia. *Gerontology* **2009**, *55*, 106–113, doi:10.1159/000176300.

[193] Rettke, H.; Geschwindner, H.M. Long-term outcomes of stroke rehabilitation: patients and informal caregivers. *Pflege* **2014**, *27*, 131–133, doi:10.1024/1012-5302/a000353.

[198] Edmonds, P.; Hart, S.; Wei, G.; Vivat, B.; Burman, R.; Silber, E.; Higginson, I.J. Palliative care for people severely affected by multiple sclerosis: evaluation of a novel palliative care service. *Multiple sclerosis* **2010**, *16*, 627–636, doi:10.1177/1352458510364632.

[235] Campbell, P.; Wright, J.; Oyebode, J.; Job, D.; Crome, P.; Bentham, P.; Jones, L.; Lendon, C. Determinants of burden in those who care for someone with dementia. *International Journal of Geriatric Psychiatry* **2008**, *23*, 1078–1085, doi:10.1002/gps.2071.

[246] Gratao, A.C.M.; Vendrúscolo, T.R.P.; Talmelli, Luana Flávia da Silva; Figueiredo, L.C.; Santos, J.L.F.; Rodrigues, R.A.P. Sobrecarga e desconforto emocional em cuidadores de idosos. *Enfermagem* **2012**, *21*, 304–312.

[256] Hu, X.; Peng, X.; Su, Y.; Huang, W. Caregiver burden among Chinese family caregivers of patients with lung cancer: A cross-sectional survey. *European Journal of Oncology Nursing* **2018**, *37*, 74–80, doi:10.1016/j.ejon.2018.11.003.

[258] Aidants proches Bruxelles. *Outils pour les aidants proches: L´ échelle de Zarit*. Retrieved from www.aidantsproches.brussels/echelle-de-zarit

[259] Akkuş, Y. Multiple sclerosis patient caregivers: the relationship between their psychological and social needs and burden levels. *Disability and Rehabilitation* **2011**, *33*, 326–333, doi:10.3109/09638288.2010.490866.

[260] Akpan-Idiok, P.A.; Anarado, A.N. Perceptions of burden of caregiving by informal caregivers of cancer patients attending University of Calabar Teaching Hospital, Calabar, Nigeria. *The Pan African Medical Journal* **2014**, *18*, 159, doi:10.11604/pamj.2014.18.159.2995.

[261] Ankri, J.; Andrieu, S.; Beaufils, B.; Grand, A.; Henrard, J.C. Beyond the global score of the Zarit Burden Interview: useful dimensions for clinicians. *International Journal of Geriatric Psychiatry* **2005**, *20*, 254–260, doi:10.1002/gps.1275.

[262] Arai, A.; Matsumoto, T.; Ikeda, M.; Arai, Y. Do family caregivers perceive more difficulty when they look after patients with early onset dementia compared to those with late onset dementia? *International Journal of Geriatric Psychiatry* **2007**, *22*, 1255–1261, doi:10.1002/gps.1935.
[263] Arai, Y. Family caregiver burden in the context of the long-term care insurance system. *Journal of Epidemiology* **2004**, *14*, 139–142, doi:10.2188/jea.14.139.
[264] Arai, Y.; Kumamoto, K.; Washio, M.; Ueda, T.; Miura, H.; Kudo, K. Factors related to feelings of burden among caregivers looking after impaired elderly in Japan under the Long-Term Care insurance system. *Psychiatry and Clinical Neurosciences* **2004**, *58*, 396–402, doi:10.1111/j.1440-1819.2004.01274.x.
[265] Arai, Y.; Sugiura, M.; Washio, M.; Miura, H.; Kudo, K. Caregiver depression predicts early discontinuation of care for disabled elderly at home. *Psychiatry and Clinical Neurosciences* **2001**, *55*, 379–382, doi:10.1046/j.1440-1819.2001.00878.x.
[266] Arai, Y.; Kudo, K.; Hosokawa, T.; Washio, M.; Miura, H.; Hisamichi, S. Reliability and validity of the Japanese version of the Zarit Caregiver Burden interview. *Psychiatry and Clinical Neurosciences* **1997**, *51*, 281–287, doi:10.1111/j.1440-1819.1997.tb03199.x.
[267] Braun, M.; Scholz, U.; Hornung, R.; Martin, M. Caregiver burden with dementia patients. A validation study of the German language version of the Zarit Burden Interview. *Zeitschrift für Gerontologie und Geriatrie* **2010**, *43*, 111–119, doi:10.1007/s00391-010-0097-6.
[268] Braun, M.; Scholz, U.; Hornung, R.; Martin, M. The burden of spousal caregiving: a preliminary psychometric evaluation of the German version of the Zarit burden interview. *Aging & Mental Health* **2010**, *14*, 159–167, doi:10.1080/13607860802459781.
[269] Brink, P.; Stones, M.; Smith, T.F. Confirmatory factor analysis of the burden interview of the caregivers of terminally ill home care clients. *Journal of Palliative Medicine* **2012**, *15*, 967–970, doi:10.1089/jpm.2012.0086.
[270] Canonici, A.P.; Andrade, L.P. de; Gobbi, S.; Santos-Galduroz, R.F.; Gobbi, L.T.B.; Stella, F. Functional dependence and caregiver burden in Alzheimer's disease: a controlled trial on the benefits of motor intervention. *Psychogeriatrics* **2012**, *12*, 186–192, doi:10.1111/j.1479-8301.2012.00407.x.
[271] Caqueo-Urizar, A.; Gutierrez-Maldonado, J. Burden of care in families of patients with schizophrenia. *Quality of Life Research* **2006**, *15*, 719–724, doi:10.1007/s11136-005-4629-2.
[272] Carretero, S.; Garces, J.; Rodenas, F. Evaluation of the home help service and its impact on the informal caregiver's burden of dependent elders. *International Journal of Geriatric Psychiatry* **2007**, *22*, 738–749, doi:10.1002/gps.1733.
[273] Chattat, R.; Cortesi, V.; Izzicupo, F.; Del Re, M.L.; Sgarbi, C.; Fabbo, A.; Bergonzini, E. The Italian version of the Zarit Burden Interview: a validation study. *International Psychogeriatrics* **2011**, *23*, 797–805, doi:10.1017/s1041610210002218.
[274] Choo, W.-Y.; Low, W.-Y.; Karina, R.; Poi, P.J.H.; Ebenezer, E.; Prince, M.J. Social support and burden among caregivers of patients with dementia in Malaysia. *Asia-Pacific Journal of Public Health* **2003**, *15*, 23–29, doi:10.1177/101053950301500105.
[275] Cifu, D.X.; Carne, W.; Brown, R.; Pegg, P.; Ong, J.; Qutubuddin, A.; Baron, M.S. Caregiver distress in parkinsonism. *Journal of Rehabilitation Research and Development* **2006**, *43*, 499–508, doi:10.1682/jrrd.2005.08.1365.
[276] Cliche, M.; Tremblay, J. *Dépistagedes besoinsdes prochesaidantsà l'aided'outils: Conférence présentée dans le cadre du colloque Initiative ministérielle sur la maladie d'Alzheimer;* Centre integre universitaire de sante et de services sociaux de la Capitale-Nationale - CIUSSS, 2016.
[277] Coen, R.F.; O'Boyle, C.A.; Coakley, D.; Lawlor, B.A. Dementia carer education and patient behaviour disturbance. *International Journal of Geriatric Psychiatry* **1999**, *14*, 302–306.
[278] Colantonio, A.; Kositsky, A.J.; Cohen, C.; Vernich, L. What support do caregivers of elderly want?: Results from the Canadian Study of Health and Aging. *Canadian Journal of Public Health/ Revue canadienne de sante publique* **2001**, *92*, 376–379.
[279] Colvez, A.; Joel, M.-E.; Ponton-Sanchez, A.; Royer, A.-C. Health status and work burden of Alzheimer patients' informal caregivers: comparisons of five different care programs in the European Union. *Health Policy* **2002**, *60*, 219–233, doi:10.1016/s0168-8510(01)00215-9.
[280] Costa-Requena, G.; Cristófol, R.; Cañete, J. Caregivers' morbidity in palliative care unit: predicting by gender, age, burden and self-esteem. *Supportive Care in Cancer* **2012**, *20*, 1465–1470, doi:10.1007/s00520-011-1233-6.
[281] Cox, C.; Monk, A. Strain among caregivers: comparing the experiences of African American and Hispanic caregivers of Alzheimer's relatives. *International Journal of Aging & Human Development* **1996**, *43*, 93–105, doi:10.2190/dyq1-tprp-vhtc-38vu.
[282] Crespo, M.; Fernandez-Lansac, V. Factors associated with anger and anger expression in caregivers of elderly relatives. *Aging & Mental Health* **2014**, *18*, 454–462, doi:10.1080/13607863.2013.856857.
[283] Czaja, S.J.; Gitlin, L.N.; Schulz, R.; Zhang, S.; Burgio, L.D.; Stevens, A.B.; Nichols, L.O.; Gallagher-Thompson, D. Development of the risk appraisal measure: a brief screen to identify risk areas and guide interventions for dementia caregivers. *Journal of the American Geriatrics Society* **2009**, *57*, 1064–1072, doi:10.1111/j.1532-5415.2009.02260.x.
[284] Dayapoğlu, N.; Tan, M. The care burden and social support levels of caregivers of patients with multiple sclerosis. *Kontakt* **2017**, *19*, e17-e23, doi:10.1016/j.kontakt.2016.12.001.
[285] Dias, A.; Dewey, M.E.; D'Souza, J.; Dhume, R.; Motghare, D.D.; Shaji, K.S.; Menon, R.; Prince, M.; Patel, V. The effectiveness of a home care program for supporting caregivers of persons with dementia in developing countries: a randomised controlled trial from Goa, India. *PloS one* **2008**, *3*, e2333, doi:10.1371/journal.pone.0002333.
[286] Edwards, N.E.; Scheetz, P.S. Predictors of burden for cregivers of patients with Parkinson's disease - ProQuest. *Journal of Neuroscience Nursing* **2002**, *34*, 184–190.
[287] Epstein-Lubow, G.; Davis, J.D.; Miller, I.W.; Tremont, G. Persisting burden predicts depressive symptoms in dementia caregivers. *Journal of Geriatric Psychiatry and Neurology* **2008**, *21*, 198–203, doi:10.1177/0891988708320972.

[288] Ezzat, O. Quality of Life and Subjective Burden on Family Caregiver of Children with Autism. *American Journal of Nursing Science* **2017**, *6*, 33, doi:10.11648/j.ajns.20170601.15.

[289] Faison, K.J.; Faria, S.H.; Frank, D. Caregivers of chronically ill elderly: perceived burden. *Journal of Community Health Nursing* **1999**, *16*, 243–253, doi:10.1207/s15327655jchn1604_4.

[290] Ferri, C.P.; Schoenborn, C.; Kalra, L.; Acosta, D.; Guerra, M.; Huang, Y.; Jacob, K.S.; Llibre Rodriguez, J.J.; Salas, A.; Sosa, A.L.; et al. Prevalence of stroke and related burden among older people living in Latin America, India and China. *Journal of Neurology, Neurosurgery, and Psychiatry* **2011**, *82*, 1074–1082, doi:10.1136/jnnp.2010.234153.

[291] Fialho, P.P.A.; Koenig, A.M.; Dos Santos, E.L.; Guimarães, H.C.; Beato, R.G.; Carvalho, V.A.; Machado, T.H.; Caramelli, P. Dementia caregiver burgen in a brasilian sample: Association to neuropsychiatric symptoms. *Dementia & Neuropsychologia* **2009**, *3*, 132–135, doi:10.1590/s1980-57642009dn30200011.

[292] Freeman, S.; Kurosawa, H.; Ebihara, S.; Kohzuki, M. Caregiving burden for the oldest old: a population based study of centenarian caregivers in Northern Japan. *Archives of Gerontology and Geriatrics* **2010**, *50*, 282–291, doi:10.1016/j.archger.2009.04.008.

[293] Fried, T.R.; Bradley, E.H.; O'Leary, J.R.; Byers, A.L. Unmet desire for caregiver-patient communication and increased caregiver burden. *Journal of the American Geriatrics Society* **2005**, *53*, 59–65, doi:10.1111/j.1532-5415.2005.53011.x.

[294] Frisbee, K.L. The impact of mobile health (mhealth) technology on family caregiver's burden levels and an assessment of variations in mhealth tool use - ProQuest; University, George Washington, 2015.

[295] Frischknecht, E. Oscar Online-Coaching. Entwicklung und erste Evaluation eines Internet-basierten Unterstützungsprogramms für Angehörige von Menschen mit einer im Erwachsenenalter erworbenen Hirnverletzung; Bern, Universität, 2011.

[296] Gallicchio, L.; Siddiqi, N.; Langenberg, P.; Baumgarten, M. Gender differences in burden and depression among informal caregivers of demented elders in the community. *International Journal of Geriatric Psychiatry* **2002**, *17*, 154–163, doi:10.1002/gps.538.

[297] Gater, A.; Rofail, D.; Marshall, C.; Tolley, C.; Abetz-Webb, L.; Zarit, S.H.; Berardo, C.G. Assessing the Impact of Caring for a Person with Schizophrenia: Development of the Schizophrenia Caregiver Questionnaire. *The Patient - Patient-Centered Outcomes Research* **2015**, *8*, 507–520, doi:10.1007/s40271-015-0114-3.

[298] Girardi Paskulin, L.M.; Kottwitz Bierhals, Carla Cristiane Becker; Oliveira Dos Santos, N.; Baltar Day, C.; Oliveira Machado, D. de; Pinheiro de Morais, E.; Rodrigues Goncalves, M.; Peixoto Cordova, F.; Ferreira Grillo, Maria de Fatima. Depressive symptoms of the elderly people and caregiver's burden in home care. *Investigacion y educacion en enfermeria* **2017**, *35*, 210–220, doi:10.17533/udea.iee.v35n2a10.

[299] Gonyea, J.G.; O'Connor, M.; Carruth, A.; Boyle, P.A. Subjective appraisal of Alzheimer's disease caregiving: the role of self-efficacy and depressive symptoms in the experience of burden. *American Journal of Alzheimer's Disease and other Dementias* **2005**, *20*, 273–280, doi:10.1177/153331750502000505.

[300] Göriş, S.; Klç, Z.; Elmal, F.; Tutar, N.; Takc, Ö. Care Burden and Social Support Levels of Caregivers of Patients with Chronic Obstructive Pulmonary Disease. *Holistic Nursing Practice* **2016**, *30*, 227–235, doi:10.1097/hnp.0000000000000153.

[301] Gort, A.M.; Mingot, M.; Gomez, X.; Soler, T.; Torres, G.; Sacristan, O.; Miguelsanz, S.; Nicolas, F.; Perez, A.; Miguel, M. de; et al. Use of the Zarit scale for assessing caregiver burden and collapse in caregiving at home in dementias. *International Journal of Geriatric Psychiatry* **2007**, *22*, 957–962, doi:10.1002/gps.1770.

[302] Gratão, A.C.M.; Brigola, A.G.; Ottaviani, A.C.; Luchesi, B.M.; Souza, É.N.; Rossetti, E.S.; Oliveira, N.A. de; Terassi, M.; Pavarini, S.C.I. Brief version of Zarit Burden Interview (ZBI) for burden assessment in older caregivers. *Dementia & Neuropsychologia* **2019**, *13*, 122–129.

[303] Grunfeld, E.; Coyle, D.; Whelan, T.; Clinch, J.; Reyno, L.; Earle, C.C.; Willan, A.; Viola, R.; Coristine, M.; Janz, T.; et al. Family caregiver burden: results of a longitudinal study of breast cancer patients and their principal caregivers. *CMAJ : Canadian Medical Association journal = journal de l'Association medicale canadienne* **2004**, *170*, 1795–1801, doi:10.1503/cmaj.1031205.

[304] Higginson, I.J.; Gao, W. Caregiver assessment of patients with advanced cancer: concordance with patients, effect of burden and positivity. *Health and Quality of Life Outcomes* **2008**, *6*, 42, doi:10.1186/1477-7525-6-42.

[305] Higginson, I.J.; Gao, W.; Jackson, D.; Murray, J.; Harding, R. Short-form Zarit Caregiver Burden Interviews were valid in advanced conditions. *Journal of Clinical Epidemiology* **2010**, *63*, 535–542, doi:10.1016/j.jclinepi.2009.06.014.

[306] Imarhiagbe, F.A.; Asemota, A.U.; Oripelaye, B.A.; Akpekpe, J.E.; Owolabi, A.A.; Abidakun, A.O.; Akemokwe, F.M.; Ogundare, V.O.; Azeez, A.L.; Osakue, J.O. Burden of informal caregivers of stroke survivors: Validation of the Zarit burden interview in an African population. *Annals of African Medicine* **2017**, *16*, 46–51, doi:10.4103/aam.aam_213_16.

[307] Kahriman, F.; Zaybak, A. Caregiver Burden and Perceived Social Support among Caregivers of Patients with Cancer. *Asian Pacific Journal of Cancer Prevention* **2015**, *16*, 3313–3317, doi:10.7314/apjcp.2015.16.8.3313.

[308] Kühnel, M.B.; Ramsenthaler, C.; Bausewein, C.; Fegg, M.; Hodiamont, F. Validation of two short versions of the Zarit Burden Interview in the palliative care setting: a questionnaire to assess the burden of informal caregivers. *Supportive Care in Cancer* **2020**, doi:10.1007/s00520-019-05288-w.

[309] Kumamoto, K.; Arai, Y.; Zarit, S.H. Use of home care services effectively reduces feelings of burden among family caregivers of disabled elderly in Japan: preliminary results. *International Journal of Geriatric Psychiatry* **2006**, *21*, 163–170, doi:10.1002/gps.1445.

[310] Lim, Y.M.; Ahn, Y.-H. Burden of family caregivers with schizophrenic patients in Korea. *Applied Nursing Research* **2003**, *16*, 110–117, doi:10.1016/S0897-1897(03)00007-7.

[311] Liu, Z.; Albanese, E.; Li, S.; Huang, Y.; Ferri, C.P.; Yan, F.; Sousa, R.; Dang, W.; Prince, M. Chronic disease prevalence and care among the elderly in urban and rural Beijing, China - a 10/66 Dementia Research Group cross-sectional survey. *BMC Public Health* **2009**, *9*, 145, doi:10.1186/1471-2458-9-394.
[312] Machnicki, G.; Allegri, R.F.; Dillon, C.; Serrano, C.M.; Taragano, F.E. Cognitive, functional and behavioral factors associated with the burden of caring for geriatric patients with cognitive impairment or depression: evidence from a South American sample. *International Journal of Geriatric Psychiatry* **2009**, *24*, 382–389, doi:10.1002/gps.2133.
[313] Mahinda, L.W. Evaluation of the informal caregiver burden in the care of stroke patients at Kenyatta national hospital. Masterarbeit Medizin; University of Nairobi.
[314] Manso Martíez, M.E.; Sáchez Lóez, Mª del Pilar; Flores, I.C. Salud y sobrecarga percibida en personas cuidadoras familiares de una zona rural. *Clínica y Salud* **2013**, *24*, 37–45, doi:10.5093/cl2013a5.
[315] Martín-Carrasco, M.; Martín, M.F.; Valero, C.P.; Millán, P.R.; García, C.I.; Montalbán, S.R.; Vázquez, A.L.G.; Piris, S.P.; Vilanova, M.B. Effectiveness of a psychoeducational intervention program in the reduction of caregiver burden in alzheimer's disease patients' caregivers. *International Journal of Geriatric Psychiatry* **2009**, *24*, 489–499, doi:10.1002/gps.2142.
[316] Maseda, A.; González-Abraldes, I.; Labra, C. de; Marey-López, J.; Sánchez, A.; Millán-Calenti, J.C. Risk Factors of High Burden Caregivers of Dementia Patients Institutionalized at Day-Care Centres. *Community Mental Health Journal* **2015**, *51*, 753–759, doi:10.1007/s10597-014-9795-7.
[317] Miyamoto, Y.; Ito, H.; Otsuka, T.; Kurita, H. Caregiver burden in mobile and non-mobile demented patients: a comparative study. *International Journal of Geriatric Psychiatry* **2002**, *17*, 765–773, doi:10.1002/gps.694.
[318] Mohamed, S.; Rosenheck, R.; Lyketsos, C.G.; Schneider, L.S. Caregiver Burden in Alzheimer Disease: Cross-Sectional and Longitudinal Patient Correlates. *The American Journal of Geriatric Psychiatry* **2010**, *18*, 917–927, doi:10.1097/JGP.0b013e3181d5745d.
[319] Nerich, V. *Protocole de Recherche. Cohorte prospective multicentrique d'aidants informels en Bourgogne Franche-Comté. Cohorte ICE (Informal Carers of Elderly)*, 2018 (21.05.2020).
[320] Ng, A.Y.M.; Wong, F.K.Y. Effects of a Home-Based Palliative Heart Failure Program on Quality of Life, Symptom Burden, Satisfaction and Caregiver Burden: A Randomized Controlled Trial. *Journal of Pain and Symptom Management* **2018**, *55*, 1–11, doi:10.1016/j.jpainsymman.2017.07.047.
[321] Ong, H.L.; Vaingankar, J.A.; Abdin, E.; Sambasivam, R.; Fauziana, R.; Tan, M.-E.; Chong, S.A.; Goveas, R.R.; Chiam, P.C.; Subramaniam, M. Resilience and burden in caregivers of older adults: moderating and mediating effects of perceived social support. *BMC Psychiatry* **2018**, *18*, 374, doi:10.1186/s12888-018-1616-z.
[322] O'Rourke, N.; Tuokko, H.A. Psychometric Properties of an Abridged Version of the Zarit Burden Interview Within a Representative Canadian Caregiver Sample. *The Gerontologist* **2003**, *43*, 121–127, doi:10.1093/geront/43.1.121.
[323] Orueta-Sánchez, R.; Gómez-Calcerrada, R.M.; Gómez-Caro, S.; Sánchez-Oropesa, A.; López-Gil, M.J.; Toledano-Sierra, P. Impacto sobre el cuidador principal de una intervención realizada a personas mayores dependientes. *Atención Primaria* **2011**, *43*, 490–496, doi:10.1016/j.aprim.2010.09.011.
[324] Pallant, J.F.; Reid, C. Measuring the positive and negative aspects of the caring role in community versus aged care setting. *Australasian Journal on Ageing* **2014**, *33*, 244–249, doi:10.1111/ajag.12046.
[325] Papastavrou, E.; Kalokerinou, A.; Papacostas, S.S.; Tsangari, H.; Sourtzi, P. Caring for a relative with dementia: family caregiver burden. *Journal of Advanced Nursing* **2007**, *58*, 446–457, doi:10.1111/j.1365-2648.2007.04250.x.
[326] Penning, M.J. Cognitive Impairment, Caregiver Burden, and the Utilization of Home Health Services. *Journal of Aging and Health* **1995**, *7*, 233–253, doi:10.1177/089826439500700204.
[327] Pérez, J.J.N.; Marqués, Á.C. Sobrecarga familiar, apoyo social y salud comunitaria en cuidadores de personas con trastorno mental grave. *Revista da Escola de Enfermagem da USP* **2018**, *52*, 31, doi:10.1590/s1980-220x2017029403351.
[328] Perrin, P.B.; Panyavin, I.; Morlett Paredes, A.; Aguayo, A.; Macias, M.A.; Rabago, B.; Picot, S.J.F.; Arango-Lasprilla, J.C. A Disproportionate Burden of Care: Gender Differences in Mental Health, Health-Related Quality of Life, and Social Support in Mexican Multiple Sclerosis Caregivers. *Behavioural Neurology* **2015**, *2015*, 1–9, doi:10.1155/2015/283958.
[329] République et canton de Genève. *Testez votre épuisement, évaluez vos limites: la mini-grille Zarit* (01.05.2020). Available online: https://www.ge.ch/testez-votre-epuisement-evaluez-vos-limites.
[330] Scazufca, M. Brazilian version of the Burden Interview scale for the assessment of burden of care in carers of people with mental illnesses. *Psychological Medicine* **2002**, *24*, 12–17, doi:10.1590/S1516-44462002000100006.
[331] Schoenmakers, B.; Buntinx, F.; DeLepeleire, J. Supporting the dementia family caregiver: The effect of home care intervention on general well-being. *Aging & Mental Health* **2010**, *14*, 44–56, doi:10.1080/13607860902845533.
[332] Sono, T.; Oshima, I.; Ito, J. Family needs and related factors in caring for a family member with mental illness: Adopting assertive community treatment in Japan where family caregivers play a large role in community care. *Psychiatry and Clinical Neurosciences* **2008**, *62*, 584–590, doi:10.1111/j.1440-1819.2008.01852.x.
[333] Sutcliffe, C.; Giebel, C.; Bleijlevens, M.; Lethin, C.; Stolt, M.; Saks, K.; Soto, M.E.; Meyer, G.; Zabalegui, A.; Chester, H.; et al. Caring for a Person With Dementia on the Margins of Long-Term Care: A Perspective on Burden From 8 European Countries. *Journal of the American Medical Directors Association* **2017**, *18*, 967-973.e1, doi:10.1016/j.jamda.2017.06.004.
[334] Taub, A.; Andreoli, S.B.; Bertolucci, P.H. Dementia caregiver burden: reliability of the Brazilian version of the Zarit caregiver burden interview. *The Gerontologist* **2004**, *20*, 372–376, doi:10.1590/S0102-311X2004000200004.
[335] Université de Sherbrooke. Être aidant pas si évident! Guide de prévention de l'épuisement pour les aidants naturels **2008**.

[336] Work + Care. *Inventaire du fardeau du proche aidant (ou Echelle de Zarit)*, 2015. Available online: http://www.info-workcare.ch/sites/default/files/documents/inventaire_du_fardeau_du_proche_aidant.pdf.
