# Supplementary material for: Self-Assessment Instruments for Supporting Family Caregivers: An Integrative Review
Source: Healthcare (Basel). 2024 May 14;12(10):1016. doi: 10.3390/healthcare12101016 (PMC11120749; doi:10.3390/healthcare12101016)
Supplement: Supplementary file 1 [file healthcare-12-01016-s001.zip › healthcare-2935572-supplementary_3.pdf]

healthcare

MDPI

Supplementary File S3: Result vignettes for self-assessment instruments

Table of contents

Burden Scale for Family Caregivers—BSFC/ Home Care Scale–HPS [34] 2

Caregiver Burden Inventory–CBI [36] 4

Caregiver Self Assessment Questionnaire—CSAQ [42] 7

Caregiver Strain Index—CSI [43] 10

Caregiving Appraisal Scale—CAS [46] 12

Caregiving Hassles Scale—CHS [48] 15

Caregiving Health Engagement Scale—CHE-s [49] 17

Carer's Checklist [54] 19

Zarit Burden Interview—ZBI [76] 21

Notes

We hereby expressly distance ourselves from all contents of all linked page addresses in this report. The operators themselves are responsible for the contents of the linked page addresses. The content was translated from German into English using an online tool and checked by hand. We apologize for any inconsistencies.

Introduction

The vignettes each comprise two pages, with the part with a blue background giving an overview of the self-assessment tool and the part with a grey background providing more in-depth information. The vignette is structured as follows:

- Description of the target group as stated by the developers
- Availability of the instrument in which languages
- Brief description of the content of the instrument
- Advantages and disadvantages of the instrument from the author's point of view
- Recommendations for application in the Swiss context from the author's point of view

The second page provides more detailed information on the number of questions, the structure of the scale, the score and the cut-off. The authors of the instrument are named and in which year and country the instrument was developed. This is followed by

information on existing translations and validations of the instrument. It should be noted that the information is only given for the relevant languages German, French, Italian and English. Subsequently, existing short versions or modifications of the instrument are named, possible target groups and information on the availability of the instrument are given. Here, references to authorship and whether digital versions of the instrument are available are given. Finally, there are references to literature. For further information on recommendations, on the summary of the instruments found and the conclusions derived from them, the results report provides information.

**Burden Scale for Family Caregivers - BSFC/ Home Care Scale – HPS [34]**	
**RECOMMENDATION FOR USE WITH REGARD TO**	
**TARGET GROUP**	Adult, caring relatives who care for a person with chronic assistance or care needs (e.g. personal care and any kind of support) in the home environment.
**LANGUAGE**	☒ German ☒ French ☒ Italian ☒ English
**CONTENT DESCRIPTION**	The instrument covers 5 different areas.<br>- Consumption of mental and physical energy<br>- Excessive demand<br>- Deterioration of the socio-economic situation<br>- Tensions in the relationship between the caring relatives and the cared-for persons<br>- Identification problems with the role of family caregivers
**ADVANTAGES (+) AND DISADVANTAGES (-) OF THE INSTRUMENT**	+ The BSFC/ HPS is easy to understand and can be completed independently by family caregivers (5-10 minutes).<br>+ The BSFC/ HPS is also available in a short version (BFSC-s) with ten questions.<br>+ The BSFC/ HPS calculates a sum value and is a valid measure of the total burden of caring relatives.<br>+ The BSFC/ HPS is suitable for use in practice and in research.<br><br>- There is no web-based version available yet.
**CONCLUSION FOR APPLICATION IN NURSING PRACTICE**	
**RECOMMENDATION**	- Using the BSFC/ HPS helps to reflect on what the family support network looks like, what can be improved and what support services provided by professionals can bring relief to their situation.<br>- The BSFC/ HPS is suitable for independent use by caring relatives. The questionnaire is available as a paper version with a template for calculating the sum value and thus facilitates the calculation of the total burden.<br>- Application by parents of sick and impaired children is possible.<br>- A digitalized version would enable the automatic calculation of the total load. This requires clarification of the possibilities of use with the developers.

	- The BSFC/ HPS (long version and short version) is available in English, German, French and Italian.

Supplementary information on the Burden Scale for Family Caregivers - BSFC/ Home Care Scale - HPS		
**Number of questions**	Long version: 28; Short version: 10	
**Scale, Score, Cut-off Score**	The statements to be assessed in the questionnaire refer to the type of help caring relatives give. This can be support, care or nursing. An overall score can be calculated.<br>The questions are scored on a scale from zero *("strongly disagree")* to three *("strongly agree")*. The overall score ranges from zero to 84 points. Higher scores indicate a greater burden on the family caregivers.	
**DEVELOPMENT**		
**Authors**	E. Grässel, and M. Leutbecher [34]	
**Year**	1993	
**Country/ Language**	Germany/ German	
**LANGUAGES**		
**Translation/ Country**	☒ English<br>☒ German<br>☒ French<br>☒ Italian	http://www.caregiver-burden.eu
**Validation**	☒ German	[113]
	☐ French	
	☐ Italian	
	☒ English	[108]
**Modified version**	Short Version	[106]
**FOR TARGET GROUP:**		
Family caregivers of persons with	Dementia disease in the home setting [34]<br>Chronic diseases and chronic care needs [107,117].<br>Amyotrophic lateral sclerosis - ALS [114]	
**AVAILABILITY OF THE INSTRUMENT**		
**Paper Pencil and/ or digital version**	☒ Paper Pencil, PDF for printing and set with test material	
**Transferability**	A manual (in German and English) is available for the introduction of BSFC/ HFS [108,34]	
**Access online version**	PDF for printing: Free version in German, English, French and Italian: [109]	

UNDERLYING THEORETICAL MODELS	Model of subjective stress and associated aspects [108]
CONTACT	http://www.caregiver-burden.eu
LITERATURE	
34. Grässel, E.; Leutbecher, M. Häusliche Pflege-Skala: HPS ; zur Erfassung der Belastung bei betreuenden oder pflegenden Personen; Vless: Ebersberg, 1993; p. 52.<br>106. Grässel E.; Berth, H.; Lichte, T.; Grau, H. Subjective caregiver burden: validity of the 10-item short version of the Burden Scale for Family Caregivers BSFC-s. BMC Geriatrics 2014, 14, 23, doi:10.1186/1471-2318-14-23.<br>107. Grässel, E. Somatic symptoms and caregiving strain among family caregivers of older patients with progressive nursing needs. Archives of Gerontology and Geriatrics 1995, 21, 253–266, doi:10.1016/0167-4943(95)00660-d.<br>108. Grässel E.; Chiu, T.; Oliver, R. Development and Validation of the Burden Scale for Family Caregivers (BSFC); Comprehensive Rehabilitation and Mental Health Services: Toronto, 2003.<br>109. Grässel, E. "Burden Scale for Family Caregivers" in 20 Sprachen, o.J (02.05.2020). Available online: www.caregiver-burden.eu.<br>113. Grau, H.; Graessel, E.; Berth, H. The subjective burden of informal caregivers of persons with dementia: extended validation of the German language version of the Burden Scale for Family Caregivers (BSFC). Aging & Mental Health 2015, 19, 159–168, doi:10.1080/13607863.2014.920296.<br>114. Hecht, M.J.; Graesel, E.; Tigges, S.; Hillemacher, T.; Winterholler, M.; Hilz, M.-J.; Heuss, D.; Neundörfer, B. Burden of care in amyotrophic lateral sclerosis. Palliative Medicine 2003, 17, 327–333, doi:10.1191/0269216303pm754oa.<br>117. Pendergrass, A.; Malnis, C.; Graf, U.; Engel, S.; Graessel, E. Screening for caregivers at risk: Extended validation of the short version of the Burden Scale for Family Caregivers (BSFC-s) with a valid classification system for caregivers caring for an older person at home. BMC Health Services Research 2018, 18, 76, doi:10.1186/s12913-018-3047-4.	

Caregiver Burden Inventory – CBI [36]	
RECOMMENDATION FOR USE WITH REGARD TO	
TARGET GROUP	The CBI is suitable for use by adult caregivers of any age (family members and friends) of cognitively impaired persons living at home. The developers of the instrument distinguish whether caring relatives live in the same household or in different households.
LANGUAGE	☐ German ☐ French ☒ Italian ☒ English
CONTENT DESCRIPTION	Includes five question areas:<br>- Questions (5) on time-dependent burden of caregiving tasks (time expenditure, e.g. *"my .... needs my help in doing many daily tasks"*).<br>- Questions (5) about developmental stress (e.g. *"I feel like I'm missing out on life"*).<br>- Questions (4) about physical stress (e.g. *"I don't sleep enough."*)<br>- Questions (5) about social stress (e.g. *"I hold a grudge against other relatives who could help but don't"*).<br>- Questions (5) about emotional distress (e.g. *"I resent my relative in need of care"*).
ADVANTAGES (+) AND	+ It quantifies stresses in different areas of the lives of family caregivers.

**DISADVANTAGES (-) OF THE INSTRUMENT**	+ The CBI enables the creation and interpretation of an individual Caregiver Burden Profile (CBP).<br>+ It includes short, simple questions that can be answered independently by family caregivers. It takes 10-15 minutes to complete.<br>+ The CBI is multidimensional, meaning that it takes into account time and developmental, physical, social and emotional issues of family caregivers<br>+ From this, appropriate measures to support caring relatives can be derived and planned.<br><br>- The CBI provides suggestions for solutions, but these have not been tested for usefulness in practice.<br>- There is no web-based version available yet.
**CONCLUSION FOR APPLICATION IN NURSING PRACTICE**	
**RECOMMENDATION**	- It is recommended that family caregivers complete the CBI questionnaire on their own. The developers recommend using the CBI with the help of professionals (e.g. social counselling, initial assessment in Spitex).<br>- The derivation of appropriate support measures requires a high degree of professional and counselling competence from the nursing or social work professionals [10].<br>- The individual stress profile (evaluation and assessment of the result) can then be discussed in the context of counselling, as early as possible [10].<br>- It is advisable to use the CBI early on, e.g. when caring relatives perceive themselves in their role and inform themselves about an external support offer and then repeat it in the course. This also makes it possible to check whether and how the measures taken have an impact on the situation of the relatives.
**Supplementary information on the Caregiver Burden Inventory - CBI [36]**	
**Number of questions**	24
**Scale, Score, Cut-off Score**	Each question is given a score between 0 (*not applicable*) and 4 (*very applicable*), with higher scores indicating greater burden on the relative; there are no cut-off points for classifying burden. Therefore, total scores for dimensions one, two, four and five can range from 0 to 20. An equivalent score for physical burden is obtained by multiplying the sum of the responses by 1.25.
**DEVELOPMENT**	
**Authors**	Novak & Guest [36]
**Year**	1989
**Country/ Language**	Canada/ English

LANGUAGES<br>**Translation/ Country**	☒ English<br>☐ German<br>☐ French<br>☒ Italian	USA [121]; Canada [125]
**Validation**	☒ English<br>☐ German<br>☐ French<br>☒ Italian	Italy [49] [127]
**Adaptation:**	☒ Italian	With four question domains: psycho-physical, time-dependent, social and emotional [49] [127]
FOR TARGET GROUP:<br>Family caregivers of persons with	Dementia [129.338]<br>ALS [127,339]<br>Chronic diseases (lung disease, diabetes, etc.) [49]	
AVAILABILITY OF THE INSTRUMENT<br>**Paper Pencil and/or digital version**	☒ Paper Pencil, no PDF available for printing	
**Transferability**	Novak and Guest recommend using the CBI in different care settings (see validation), and with relatives of people with different chronic conditions.	
**Direct link to the Instrument**	There is a copyright for the use.<br>The instrument is copyrighted by Dr. Mark Novak.	
**Contact for**	Adapted Italian version: *Patrizia Mecocci, Istituto di Gerontologia e Geriatria, Perugia, Serena Barello, Università Cattolica del Sacro Cuore, Milan*	
UNDERLYING THEORETICAL MODELS	Refers to own, unpublished work [36]	
CONTACT	For the Italian version: mecocci@unipg.it; serena.barello@unicatt.it<br>For the English questionnaire, a request can be made on the Clinical Outcome Assessments (COAs) database COAs.	
LITERATURE		

36. Novak, M.; Guest, C. Application of a Multidimensional Caregiver Burden Inventory1. Gerontologist 1989, 29, 798–803. https://doi.org/10.1093/geront/29.6.798.

49. Barello, S.; Castiglioni, C.; Bonanomi, A.; Graffigna, G. The Caregiving Health Engagement Scale (CHE-s): Development and initial validation of a new questionnaire for measuring family caregiver engagement in healthcare. BMC Public Health 2019, 19, 1562. https://doi.org/10.1186/s12889-019-7743-8.

121. Caserta, M.S.; Lund, D.A.; Wright, S.D. Exploring the Caregiver Burden Inventory (CBI): further evidence for a multidimensional view of burden. International Journal of Aging & Human Development 1996, 43, 21–34, doi:10.2190/2dkf-292p-a53w-w0a8.

125. Evans, R.; Catapano, M.A.; Brooks, D.; Goldstein, R.S.; Avendano, M. Family caregiver perspectives on caring for ventilator-assisted individuals at home. Canadian Respiratory Journal 2012, 19, 373–379, doi:10.1155/2012/452898.

127. Gauthier, A.; Vignola, A.; Calvo, A.; Cavallo, E.; Moglia, C.; Sellitti, L.; Mutani, R.; Chiò, A. A longitudinal study on quality of life and depression in ALS patient-caregiver couples. Neurology 2007, 68, 923–926, doi:10.1212/01.wnl.0000257093.53430.a8.

129. Iavarone, A.; Ziello, A.R.; Pastore, F.; Fasanaro, A.M.; Poderico, C. Caregiver burden and coping strategies in caregivers of patients with Alzheimer's disease. Neuropsychiatric Disease and Treatment 2014, 10, 1407–1413, doi:10.2147/ndt.S58063.

338. Chiatti, C., Masera, F., Rimland, J. M., Cherubini, A., Scarpino, O., Spazzafumo L., & Lattanzio, F. (2013). The UP-TECH project, an intervention to support caregivers of Alzheimer's disease patients in Italy: Study protocol for a randomized controlled trial. Trials, 14, 155. **https://doi.org/10.1186/1745-6215-14-155**

339. Chiò, A.; Vignola, A.; Mastro, E.; Dei Giudici, A.; Iazzolino, B.; Calvo, A.; Moglia, C.; Montuschi, A. Neurobehavioral symptoms in ALS are negatively related to caregivers' burden and quality of life. European journal of neurology 2010, 17, 1298–1303, doi:10.1111/j.1468-1331.2010.03016.x.

Caregiver Self Assessment Questionnaire - CSAQ [42]				
RECOMMENDATION FOR USE WITH REGARD TO				
TARGET GROUP	Adults, caregivers (family members, friends, close relatives) of older persons.			
LANGUAGE	☐ German	☐ French	☒ Italian	☒ English
CONTENT DESCRIPTION	The questionnaire contains 18 questions on the following areas:<br>- Caring responsibilities (e.g. *"I felt torn between work and family responsibilities"*).<br>- Intensity of care (e.g. *"I noticed that I could not leave my relative) alone"* and *"I noticed a loss of privacy and/or time for myself"*).<br>- Stresses (e.g. *"I felt completely overwhelmed")*<br>- Positive effects of care (e.g. *"I felt useful and needed" and "I was satisfied with the support my family gave me"*).<br>- State of health (e.g. *"I had back pain")*			
ADVANTAGES (+) AND DISADVANTAGES (-) OF THE INSTRUMENT	+ An easy-to-use online questionnaire is available.<br>+ Family caregivers are encouraged to take a moment to reflect on their own wellbeing.<br>+ The questionnaire can be completed at any point during the course of a relative's care.<br>+ Before anyone completes the online questionnaire, they are advised to be sure to contact a professional if they have any concerns about the questionnaire or the results.			

	- The recommendations to the relatives given on the basis of the questionnaire assessment are quite unspecific and very generally formulated. Therefore, it makes sense to seek advice from a specialist in any case.
**CONCLUSION FOR APPLICATION IN NURSING PRACTICE**	
**RECOMMENDATION**	- The questionnaire can help caregivers to assess their own behavior and health risks with regard to stress.<br>- The short online questionnaire is suitable for independent use by family caregivers. According to the recommendations of the American Medical Association (AMA), the questionnaire can be used as a guide. The results of the questionnaire should be discussed with a doctor.<br>- The result can be discussed not only with a doctor, but also with nursing and social work professionals.<br>- The supplementary information includes the result of a fictitious caregiver who completed the online questionnaire.<br>- There is a copyright. Written permission from the Health in Aging Foundation must be obtained for use.

**Supplementary information on the Caregiver Self Assessment Questionnaire - CSAQ**	
**Number of questions**	18
**Scale, Score, Cut-off Score**	Family caregivers are asked to assess the situation over the past week with:<br>16 questions on care tasks, care intensity, burdens and positive effects of care and their state of health with either *"yes"* or *"no*",<br>1 question on the current level of stress (on a scale of 1 to 10, with 1 *"not stressful"* to 10 *"extremely stressful"*),<br>1 question on the current state of health compared to the situation one year ago (on a scale of 1 to 10, with 1 *"very healthy"* to 10 *"very sick"*).<br>A total score can be calculated to identify health risks (e.g. indications of exhaustion-depression). In the online version, the total score is calculated automatically, and caring relatives receive their result and suggestions for further action directly.<br>*Fictitious example of a family caregiver* [42]<br>The family caregiver has filled out the online questionnaire.<br>*Your automatically obtained result looks like this:*<br>Please print out your result.<br>There is a possibility that you are heavily burdened at the moment.<br>Your answers show that you have problems or challenges with 10 out of 16 questions.<br>You answered *"yes" that you feel completely overwhelmed*.<br>You answered *"yes" that* you had a *crying fit.*<br>You have rated your current stress level as 7 on a scale of 1 to 10.<br><br>*We recommend the following next steps:*

	Consider going to a health care professional for a check-up.<br>Consider some relief from caregiving (discuss with a caregiver or social worker the resources available in your community).<br>Consider joining a support group.<br><br>*This is followed by a list of valuable information for family caregivers.*	
**DEVELOPMENT**<br>**Authors**<br>**Year**<br>**Country/ Language**	 American Medical Association – AMA [42]<br>2015<br>USA	
**LANGUAGES**<br>**Translation/ Country**	☒ English<br>☐ German<br>☐ French<br>☒ Italian	
**Validation**	☒ English<br>☐ German<br>☐ French<br>☐ Italian	Valid self-assessment tool for detecting depressive symptoms in family caregivers [340]
**FOR TARGET GROUP:**<br>Family caregivers of persons with	 Older persons [42]<br>People with dementia [340]	
**AVAILABILITY OF THE INSTRUMENT**<br>**Paper Pencil and/ or digital version**	 ☒ Paper Pencil: PDF for printing<br>☒ Digital: Online questionnaire	
**Transferability**	Recommended for all adult caregivers of older people. Typically, these are relatives caring for an older person with dementia.	
**Direct link to the Instrument**	Link to the online version: Caregiver Self Assessment Questionnaire<br>Link to the Italian version (PDF): Caregiver Self Assessment Questionnaire pdf	
**Contact**	There is a copyright for the use. ©2015 Health in Aging Foundation. All rights reserved. This material may not be reproduced, displayed, modified or distributed without the express prior written permission of the copyright holder. For permission, please contact info@healthinaging.org	
**UNDERLYING THEORETICAL MODELS**	None apparent	
**CONTACT**	Health in Aging Foundation info@healthinaging.org	
**LITERATURE**		
42. American Medical Association AMA. AMA Homepage. Available online: https://www.ama-assn.org/ (accessed on 05/05/2024).		

340. Epstein-Lubow, G.; Gaudiano, B.A.; Hinckley, M.; Salloway, S.; Miller, I.W. Evidence for the validity of the American Medical Association's caregiver self-assessment questionnaire as a screening measure for depression. Journal of the American Geriatrics Society 2010, 58, 387–388, doi:10.1111/j.1532-5415.2009.02701.x.

Caregiver Strain Index – CSI [43]	
**RECOMMENDATION FOR USE WITH REGARD TO**	
**TARGET GROUP**	Adults, caregivers (family members, friends, close relatives) of older persons.
**LANGUAGE**	☐ German ☐ French ☐ Italian ☒ English
**CONTENT DESCRIPTION**	The CSI asks caregivers about objective (sleep, finances, normal routine) and subjective burdens. Caregivers are asked whether they have been affected by their role as a caregiver and whether this has placed a physical and psychological burden on them.
**ADVANTAGES (+) AND DISADVANTAGES (-) OF THE INSTRUMENT**	+ It is a short, easy-to-use instrument (13 questions) that can be used to identify stress at an early stage (e.g. shortly after taking up care tasks).<br>+ The questionnaire calculates an overall score to identify high stress.<br><br>- There is no web-based version available yet.
**CONCLUSION FOR APPLICATION IN NURSING PRACTICE**	
**RECOMMENDATION**	- The Caregiver Strain Index (CSI) and also the Modified Caregiver Strain Index (MCSI) are self-assessment tools that are easy and quick to introduce and use and give an indication of whether caregivers are experiencing high levels of strain.<br>- It is frequently used in clinical practice [341].<br>- The CSI/ MCSI can be used as a one-time screening tool to identify stress in certain at-risk populations at an early stage. This includes, for example, people with stressful care relationships or other emotional burdens.<br>- Application by parents of sick and impaired children is possible.<br>- It is recommended to use the CSI/ MCSI in a consultation with a caregiver or social work professional to assess the relationship between caregiver and cared for person.

Supplementary information on the Caregiver Strain Index - CSI		
NUMBER OF QUESTIONS	13	
SCALE, SCORE, CUT-OFF SCORE	The presence of objective and subjective stress can be answered with "yes" or "no". Each "yes" receives one point and thus a maximum score of 13. The CSI has a cut-off point: a total score of 7 or more indicates a high degree of strain.	
DEVELOPMENT		
AUTHORS	Robinson [43]	
YEAR	1983	
COUNTRY/ LANGUAGE	USA/ English	
LANGUAGES		
TRANSLATION/ COUNTRY	☒ English<br>☐ German<br>☐ French<br>☐ Italian	USA [43]
VALIDATION	☒ English<br>☐ German<br>☐ French<br>☐ Italian	[43]
MODIFIED VERSION:	☒ English	Modified Caregiver Strain Index (MCSI) [342]
FOR TARGET GROUP: FAMILY CAREGIVERS OF PERSONS WITH	Alzheimer's dementia [182]<br>Amyotrophic lateral sclerosis / motor neuron disease (ALS/ MND) [185]<br>Persons with a stroke [171]<br>Sick children [175]	
AVAILABILITY OF THE INSTRUMENT		
PAPER PENCIL AND/ OR DIGITAL VERSION	☒ Paper Pencil, PDF for printing	
TRANSFERABILITY	Suitable for different target groups, also for parents of sick and impaired children.	
DIRECT LINK TO THE INSTRUMENT	Link Caregiver Strain Index (CSI)	
UNDERLYING THEORETICAL MODELS	Stress model by Pearlin [16.17]	

CONTACT	Use is copyright of the publisher, ©The Gerontological Society of America. E-mail notification of use to: hartford.ign@nyu.eduhartford.ign@nyu.edu
LITERATURE	

43. **Robinson, B.C. Validation of a Caregiver Strain Index. Journal of gerontology 1983, 38, 344–348, doi:10.1093/geronj/38.3.344.**
171. **van Exel, N.J.A.; Scholte op Reimer, W. J. M.; Brouwer, W.B.F.; van den Berg, B.; Koopmanschap, M.A.; van den Bos, G.A. Instruments for assessing the burden of informal caregiving for stroke patients in clinical practice: a comparison of CSI, CRA, SCQ and self-rated burden. Clinical Rehabilitation 2004, 18, 203–214, doi:10.1191/0269215504cr723oa.**
175. **Bonner, M.J.; Hardy, K.K.; Guill, A.B.; McLaughlin, C.; Schweitzer, H.; Carter, K. Development and validation of the parent experience of child illness. Journal of Pediatric Psychology 2006, 31, 310–321, doi:10.1093/jpepsy/jsj034.**
182. **Diwan, S.; Hougham, G.W.; Sachs, G.A. Strain experienced by caregivers of dementia patients receiving palliative care: findings from the Palliative Excellence in Alzheimer Care Efforts (PEACE) Program. Journal of Palliative Medicine 2004, 7, 797–807, doi:10.1089/jpm.2004.7.797.**
185. **Jenkinson, C.; Fitzpatrick, R.; Swash, M.; Peto, V.; and the ALS-HPS Steering Group. The ALS Health Profile Study: quality of life of amyotrophic lateral sclerosis patients and carers in Europe. Journal of Neurology 2000, 247, 835–840, doi:10.1007/s004150070069.**
341. van Heugten, C.; Visser-Meily, A.; Post, M.; Lindeman, E. Care for carers of stroke patients: evidence-based clinical practice guidelines. J Rehabil Med 2006, 38, 153-158, doi:10.1080/16501970500441898.
342. Thornton, M.; Travis, S.S. Analysis of the reliability of the modified caregiver strain index. The journals of gerontology. Series B, Psychological sciences and social sciences 2003, 58, S127-132, doi:10.1093/geronb/58.2.s127.

Caregiving Appraisal Scale - CAS [46]	
RECOMMENDATION FOR USE WITH REGARD TO	
TARGET GROUP	Adult caregivers of older people with any kind of impairment, dementia and people at the end of life.
LANGUAGE	☐ German ☐ French ☐ Italian ☒ English
CONTENT DESCRIPTION	The CAS focuses on the subjective evaluation of care and includes:<br>the subjective burden of care (9 questions),<br>satisfaction with the support (6 questions),<br>Coping with caregiving (6 questions),<br>Requirements for care (3 questions) and<br>Impact on care (3 questions).
ADVANTAGES (+) AND DISADVANTAGES (-) OF THE INSTRUMENT	+ The questionnaire contains the subjective assessment of all aspects of care (care tasks, care intensity/care effort).<br>+ Positive and negative effects of care are examined.<br>+ The CAS has been continuously developed and the question categories and the scope of questions vary depending on the care situation and environment (setting) and the group of persons cared for. Therefore, a broad application is possible.<br><br>- There is no web-based version available yet.
CONCLUSION FOR APPLICATION IN NURSING PRACTICE	

**RECOMMENDATION**	- The CAS was exclusively used in studies and further developed. However, the developer also recommends practical use. It can be included in surveys in different settings and groups of family caregivers.<br>- From our point of view, application by parents of sick and impaired children is also possible.<br>- For the independent completion of the questionnaire by caring relatives, a digital version is needed to recode and automatically calculate the sum value (score) for the individual questionnaire areas.<br>- For the application, support from care or social work professionals is needed to calculate and assess the outcome and to plan appropriate supportive interventions.<br>- The derivation of suitable support measures requires a high degree of professional and counselling competence from the nursing or social work professionals.

**Supplementary information on the Caregiving Appraisal Scale - CAS**		
**Number of questions**	27<br>9 Questions about subjective burden of caregiving<br>6 questions on satisfaction with care<br>6 questions on coping with care (6 questions)<br>3 Questions about care/care requirements<br>3 Questions about effects on care/care	
**Scale, Score, Cut-off Score**	Question type A asks the following question: "*I would like to talk about some feelings you may have about caring for your (mother, etc.). Please tell me if you"*; with the response categories (1) "*don´t agree at all*" to (5) *"agree completely"*.<br>Question type B asks the following question: *"Tell me how often you feel each way"*; with the answer categories (1) "*never*" to (5) *"almost always"*.<br>A score can be calculated for each question area. This means at: Burden of care/care (high score is burdened); Satisfaction with care/ nursing (high score is satisfied); Coping with care (high score is good coping); Requirements for care/ nursing (high score is demanding); Impact on care (high score is unfavorable impact)	
DEVELOPMENT<br>**Authors**<br>**Year**<br>**Country/ Language**	 Lawton, M. P., Kleban, M. H., Moss, M., Rovine, M., & Glicksman, A.<br>1989 [46]<br>USA/ English	
LANGUAGES<br>**Translation/ Country**	 ☒ English<br>☐ German	 USA [46,47]

	☐ French<br>☐ Italian	
**Validation**	☒ English<br>☐ German<br>☐ French<br>☐ Italian	[343]
**Revised version:**	☒ English	[344]
FOR TARGET GROUP:<br>Family caregivers of persons with	Brain injuries [345]<br>palliative care [198]<br>Dementia [200,202]	
AVAILABILITY OF THE INSTRUMENT<br>**Paper Pencil and/ or digital version**	**☒ Paper Pencil, PDF for printing**	
**Transferability**	Suitable for assessments in different settings and groups of caregivers	
**Direct link to the Instrument**	There is a copyright of the publisher for the use.<br>The revised instrument is in the study by Lawton [344] published. Permission to use, reproduce or translate this questionnaire is required. Permission must be obtained from the respective journal publisher.	
UNDERLYING THEORETICAL MODELS	Appraisal and reappraisal framework<br>Model of caregiving dynamics [47]	
CONTACT	For the English questionnaire, a request can be made on the Clinical Outcome Assessments (COAs) database COAs.	

LITERATURE

198. **Cooper, B.; Kinsella, G.J.; Picton, C. Development and initial validation of a family appraisal of caregiving questionnaire for palliative care. Psycho-Oncol. 2006, 15, 613–622. https://doi.org/10.1002/pon.1001.**
200. **Farran, C.J.; Miller, B.H.; Kaufman, J.E.; Donner, E.; Fogg, L. Finding meaning through caregiving: development of an instrument for family caregivers of persons with Alzheimer's disease. Journal of Clinical Psychology 1999, 55, 1107–1125, doi:10.1002/(sici)1097-4679(199909)55:9<1107:aid-jclp8>3.0.co;2-v.**
202. Stolley, J.M.; Reed, D.; Buckwalter, K.C. Caregiving appraisal and interventions based on the progressively lowered stress threshold model. American Journal of Alzheimer's Disease and other Dementias 2002, 17, 110–120, doi:10.1177/153331750201700211.
343. **Struchen, M.A.; Atchison, T.B.; Roebuck, T.M.; Caroselli, J.S.; Sander, A.M. A multidimensional measure of caregiving appraisal: validation of the Caregiver Appraisal Scale in traumatic brain injury. The Journal of head trauma rehabilitation 2002, 17, 132–154, doi:10.1097/00001199-200204000-00005.**
344. **Lawton, M.P.; Moss, M.; Hoffman, C.; Perkinson, M. Two transitions in daughters' caregiving careers. Gerontologist 2000, 40, 437-448, doi:10.1093/geront/40.4.437.**
345. Albert, S.M.; Im, A.; Brenner, L.; Smith, M.; Waxman, R. Effect of a social work liaison program on family caregivers to people with brain injury. The Journal of head trauma rehabilitation 2002, 17, 175–189, doi:10.1097/00001199-200204000-00007.

Caregiving Hassles Scale - CHS [48]	
RECOMMENDATION FOR USE WITH REGARD TO	
TARGET GROUP	Adults, primary caregivers of persons diagnosed with Alzheimer's dementia and living at home.
LANGUAGE	☐ German ☐ French ☐ Italian ☒ English
CONTENT DESCRIPTION	CHS focuses on the minor everyday events, the everyday experience of caring and being cared for, and minor irritations of daily life. Such stresses can be both temporary and long-lasting, and they are seen by a caregiver as affecting their well-being. The CHS asks about the occurrence of these events and assesses the distress they cause. With the Uplift Scale also from Kinney & Stephens [32] can also be used to assess the positive impact of caregiving.<br><br>The CHS contains 5 question areas:<br>- Difficulty assisting with basic ADL<br>- Difficulties in assisting with instrumental ADLs<br>- Problems with the cognitive status of the person being cared for<br>- Problems with the behavior of the person being cared for<br>- Problems with the support network
ADVANTAGES (+) AND DISADVANTAGES (-) OF THE INSTRUMENT	+ The CHS is an instrument to determine the general level of stress among family caregivers, e.g. from specific sources of stress.<br>+ The questionnaire is very comprehensive with 42 questions or 110 questions (with Uplift Scale).<br><br>- There is no web-based version available yet.
CONCLUSION FOR APPLICATION IN NURSING PRACTICE	
RECOMMENDATION	- The CHS is a diagnosis-specific instrument. It has so far been used for relatives of persons with dementia. From our point of view, it can also be used by caring relatives of persons with other problems and parents of sick and impaired children.<br>- It would be useful to use the CHS in combination with the Uplifts Scale [32] in order to record not only the stresses but also the positive effects of the previous week's care.<br>- For the independent completion of the questionnaire by caring relatives, a digital version is needed to recode individual answers and to automatically calculate the sum value (score) for the individual questionnaire areas.<br>- The CHS/ Uplift Scale gives a detailed picture of the day-to-day care experience. It needs good support from care or social work professionals to calculate and assess the outcome and to plan appropriate supportive interventions.

	- The derivation of appropriate support measures requires a high degree of professional and counselling competence from the nursing or social work professionals.	
**Supplementary information on the Caregiving Hassles Scale - CHS**		
**Number of questions**	42, in combination with the Uplift Scale 110 questions.	
**Scale, Score, Cut-off Score**	The questionnaire asks about events that have or have not occurred in the past week. If the event occurred, then the respondents rate the extent of the stress on a 4-point scale (from (1) *"not at all"* stressful to (4) *"very stressful"*.<br>In combination with the Uplift Scale, the extent of stress or positive effect can be assessed on a 4-point scale ranging from (1) "*it was not (stressful/enriching)*" to "*very (stressful/enriching)"*.	
DEVELOPMENT<br>**Authors**<br>**Year**<br>**Country/ Language**	 Kinney, J. M., & Stephens, M. A. P. [48,346]<br>1989<br>USA	
LANGUAGES<br>**Translation/ Country**	☒ English<br>☐ German<br>☐ French<br>☐ Italian	[48]
**Validation**	☒ English<br>☐ German<br>☐ French<br>☐ Italian	[48]
FOR TARGET GROUP:<br>Family caregivers of persons with	Dementia [48,346]	
AVAILABILITY OF THE INSTRUMENT<br>**Paper Pencil and/or digital version**	☒ Paper Pencil, not available as PDF for printing	
**Transferability**	The CHS has been developed and validated for relatives of severely cognitively impaired persons. Further research is needed for practical implementation.	
**Direct link to the instrument**	There is a copyright of the publisher for the use.<br>The instrument is published in the studies by Kinney & Stephens. [48] published. Permission to use, reproduce or translate this questionnaire is required. Permission must be obtained from the respective journal publisher.	

**UNDERLYING THEORETICAL MODELS**	Burden is conceptualized as a difficulty or minor irritation of daily life. [15]
**CONTACT**	For the English questionnaire, a request can be made on the Clinical Outcome Assessments (COAs) database COAs.
**LITERATURE**	
**15. Lazarus, R. S. (1999). Stress and emotion: A new synthesis. London: Free Assoc. Books.**<br>48. Kinney, J.M.; Stephens, M.A.P. Caregiving Hassles Scale: Assessing the Daily Hassles of Caring for a Family Member With Dementia. The Gerontologist 1989, 29, 328–332, doi:10.1093/geront/29.3.328. 346.<br>346. Kinney, J.M.; Stephens, M.A.P. Hassles and uplifts of giving care to a family member with dementia. Psychology and Aging 1989, 4, 402–408, doi:10.1037/0882-7974.4.4.402.	
**Caregiving Health Engagement Scale - CHE-s [49]**	
**RECOMMENDATION FOR USE WITH REGARD TO**	
**TARGET GROUP**	Family caregivers of people with complex care needs.
**LANGUAGE**	☐ German ☐ French ☒ Italian ☒ English
**CONTENT DESCRIPTION**	The CHE-s is a short questionnaire based on the perspective and views of family caregivers. The CHE-s was developed with the involvement of family caregivers to assess the psychosocial experience of caring and family caregiver involvement. The questionnaire shows to what extent a balance is achieved between the respective caregiving tasks and general life goals. The CHE-s aims to close the gap between what caring relatives experience in their everyday caregiving and what provides them with the most support.
**ADVANTAGES (+) AND DISADVANTAGES (-) OF THE INSTRUMENT**	+ CHE-s shows that the engagement of family caregivers is a psychosocial process that results from a dynamic journey of maturation and redefinition of the role of the individual family member in the course of accompanying and caring for the cared-for person.<br>+ With the CHE-s, it is possible, on the one hand, to assess the level of commitment of family caregivers and thus, on the other hand, to better tailor supportive and educational measures to their needs.<br><br>- There is no web-based version available yet.
**CONCLUSION FOR APPLICATION IN NURSING PRACTICE**	
**RECOMMENDATION**	- The CHE-s gives a detailed picture of the day-to-day care experience and which measures offer relief.<br>- Family caregivers can answer the CHE-s questions independently.

	- The application needs good support from care or social work professionals to calculate and assess the outcome and to plan appropriate supportive interventions.<br>- The derivation of suitable support measures requires a high degree of professional and counselling competence from the nursing or social work professionals.

**Supplementary information on the Caregiving Health Engagement Scale - CHE-s**		
**Number of questions**	7	
**Scale, Score, Cut-off Score**	For each criterion (CHE1-7), caregivers can choose from four statements that best describe their current experience. Each choice theoretically corresponds to one of the positions described in the CHE model (1 = denial, 2 = overactivity, 3 = overload, 4 = balance). Lower scores correspond to the "denial" and "overactivity" positions of the CHE model, higher scores to the "overload" and "balance" positions. The results indicate how the stressful situation takes shape, e.g. how the caregiver behaves and feels in the situation.<br>Further details about the scoring system are available from the authors on request.	
DEVELOPMENT<br>**Authors**<br>**Year**<br>**Country/ Language**	 Barello, Castiglioni, Bonanomi & Graffigna [49]<br>2019<br>Italy/ Italian	
LANGUAGES<br>**Translation/ Country**	☐ English<br>☐ German<br>☐ French<br>☒ Italian	[49]
**Validation**	☐ German<br>☐ French<br>☒ Italian<br>☐ English	Italy [49]
**FOR TARGET GROUP: Family caregivers of persons with**	Complex care needs [49]	
**AVAILABILITY OF THE INSTRUMENT**	☒ Paper Pencil, only available in the study, access via the author (S. Barello) of the study.	

**Paper Pencil and/or digital version**<br><br>**Transferability**<br><br>**Direct link to the instrument**<br><br>**Contact**	To adult caregivers of people with complex care needs, including caregivers of children.<br><br>Not available<br>The instrument is published in an open access format that permits unrestricted use, distribution, and reproduction in any medium, provided you give proper credit to the original author(s) and source, link to the license and whether any modifications have been made. This applies to the data provided in this article unless otherwise stated.<br><br>For the instrument: Serena Barello, Università Cattolica del Sacro Cuore, Milano
**UNDERLYING THEORETICAL MODELS**	Process of Family Caregiver Engagement described in the CHE Model [49]
**CONTACT**	serena.barello@unicatt.it
**LITERATURE**	

49. Barello, S., Castiglioni, C., Bonanomi, A., & Graffigna, G. (2019). The Caregiving Health Engagement Scale (CHE-s): Development and initial validation of a new questionnaire for measuring family caregiver engagement in healthcare. BMC Public Health, 19(1), 1562. https://doi.org/10.1186/s12889-019-7743-8

Carer's Checklist [54]

RECOMMENDATION FOR USE WITH REGARD TO

**TARGET GROUP**	Adults, caregivers (family members, friends, loved ones) of people with dementia who receive specialized professional care and by volunteers.
**LANGUAGE**	☐ German ☐ French ☐ Italian ☒ English
**CONTENT DESCRIPTION**	The Carer's Checklist for family caregivers is used<br>- to elicit the extent of dementia-related problems in daily life (objective burdens) and the resulting subjective burden.<br>- to assess the needs and changes in the needs of people with dementia and their family caregivers, and evaluate the results of measures of care.<br><br>Low Medium High<br>Scope
**ADVANTAGES (+) AND**	+ The Carer's Checklist for Family Caregivers is easy to use and takes about 15 minutes to complete independently.

Commented [DBF(1]: anpassen

**DISADVANTAGES (-) OF THE INSTRUMENT**	+ The instrument enjoys a high level of acceptance among professionals and family caregivers in its use.<br>+ The instrument is dementia-specific and should only be used in cases where there is a suspected or confirmed diagnosis of dementia.<br><br>- There is no web-based version available yet.
**CONCLUSION FOR APPLICATION IN NURSING PRACTICE**	
**RECOMMENDATION**	- Caregivers could answer the Carer's Checklist questions independently. The questions are easy to answer.<br>- In order to assess the individual stress situation (taking into account the information about the person being cared for) and to take appropriate measures, it is advisable to seek the advice of professionals.<br>- Repeating the carer checklist over time through concurrent assessments and comparing scores on assessments can provide useful information about patient and caregiver functioning over time.<br>- The calculation of the total point values can be calculated manually, with digitalization it is possible to calculate the result automatically. For practical use, a digital solution would have to be sought.<br>- Assessing the outcome and planning appropriate supportive measures requires advice from the care or social work professionals and a high level of professional and counselling competence.

**Supplementary information on the Carer's Checklist**	
**Number of questions**	35
**Scale, Score, Cut-off Score**	The Carer's Checklist consists of two parts.<br>The first part contains a list of 30 dementia-related problems that can occur. Each problem is asked and it should be ticked:<br>Objective burden: How often does the problem occur with the person being cared for?<br>0=never; 1=sometimes; 2=always, with a point maximum of 60<br>Subjective stress: How stressful do caregivers rate the problem?<br>0=not stressful; 1=quite *stressful; 2=very stressful*, with a maximum score of 60.<br>The second part of the checklist consists of five assessments (1="*not burdened at all*" to 5="*heavily burdened*") on overall burden, physical burden, financial burden, emotional burden and social burden, with a maximum score of 25.<br>The points can be calculated individually for each part and repeated over time.
**DEVELOPMENT**	

**Authors**<br>**Year**<br>**Country/ Language**	Hodgson, C.; Higginson, I.; Jefferys, P. [54]<br>1997<br>UK/ English	
LANGUAGES<br>**Translation/ Country**	☒ English<br>☐ German<br>☐ French<br>☐ Italian	[54]
**Validation**	☐ English<br>☐ German<br>☐ French<br>☒ Italian	Has not been validated in studies [54
FOR TARGET GROUP:<br>Family caregivers of persons with	People with a confirmed or suspected dementia illness [347,348]	
AVAILABILITY OF THE INSTRUMENT<br>**Paper Pencil and/or digital version**	☒ Paper Pencil, PDF for printing	
**Transferability**	Not recommended	
**Direct link to the instrument**	Link **Carer's Checklist**<br>Use must be clarified with the Mental Health Foundation UK.	
UNDERLYING THEORETICAL MODELS	--	
CONTACT	https://www.mentalhealth.org.uk/contact/form	

LITERATURE

54. Hodgson, C.; Higginson, I.; Jefferys, P. Carers Checklist; The Mental Health Foundation: London, UK, 1998.

347. Higginson, I.J.; Jefferys, P.M.; Hodgson, C.S. Outcome measures for routine use in dementia services: some practical considerations. Qual Health Care 1997, 6, 120-124, doi:10.1136/qshc.6.3.120.

348. Harvey, K.; Langman, A.; Winfield, H.; Catty, J.; Clement, S.; White, S.; Burns, E.; Burns, T. Measuring Outcomes for Carers for People with Mental Health Problems. Report for the National Coordinating Centre for NHS Service Delivery and Organisation R & D (NCCSDO); 2005.

Zarit Burden Interview - ZBI [76]

RECOMMENDATION FOR USE WITH REGARD TO				
TARGET GROUP	Adults, primary caregivers (family members and friends) of persons of all ages with various illnesses who are being cared for at home.			
LANGUAGE	☒ German	☒ French	☒ Italian	☒ English

**Content description**	The ZBI is the most frequently used instrument for assessing the subjective burden of caregivers of persons suffering from dementia. Family caregiver rates 22 statements on the behavior of the person with dementia, their own mental and physical health, financial situation, work intensity, relationship with the person with dementia and support from family members.
**Advantages (+) and disadvantages (-) of the instrument**	+ The first study by Zarit et al. (1980) found that the level of stress experienced by family caregivers of older people with dementia was not related to behavioral problems caused by the disease. Rather, the subjective burden was related to a lack of social support, especially the number of visitors at home.<br>+ Subsequent studies have described intervention methods for the family and professional support network, e.g., "network sessions", discussion groups such as self-help groups, planning of home visits by family members, friends and neighbors.<br>+ Since its development, the ZBI has been used in practice with a various target groups and care contexts and is available in many languages - including German, French and Italian.<br>+ Various validated short versions are available with 1 to 12 questions.<br><br>- It is reported from practice that relatives are sometimes frightened by the extent of the burden.<br>- It takes a medium amount of time to complete (about 25 minutes).
**CONCLUSION FOR APPLICATION IN NURSING PRACTICE**	
**Recommendation**	- Family caregivers can answer the ZBI on their own or as part of a conversation. It is advisable to seek the advice of professionals in order to assess the individual stress situation and to take appropriate measures.<br>- It is an advantage if the professionals know the instrument well. In particular, knowledge of the strong relationship between visits by family members and the burden on the primary caregiver. In this way, measures can be taken in which other relatives of the close environment of the impaired person (other family members, friends, neighborhood) are included with their resources.<br>- The ZBI should not be used as the sole indicator of the caregiver's emotional state. Clinical observations and other instruments, such as the measurement of depression, should be used in addition.<br>- It was shown that the ZBI successfully differentiates between different target groups and is suitable for measuring longitudinal developments. Application by parents of sick and impaired children is possible.<br>- Since its development, the ZBI has been used in practice for a variety of target groups and care contexts and is available in many languages - including German, French and Italian.

**Supplementary information on the Zarit Burden Interview - ZBI**	
**Number of questions**	Long version: 22; short versions: 1, 4, 7, 12

**Scale, Score, Cut-off Score**	With a short instruction, the family caregivers are asked to answer a series of questions about the impact of the illnesses of the person being cared for on their own life. For each item, they indicate how often they have felt this way (0=*never*, 1=*sometimes*, 2=*sometimes*, 3=*quite often* or 4=*almost always*).<br>The ZBI is scored by adding the numbered answers of the individual statements. Higher scores indicate greater burden on family caregivers. The levels of burden are estimates from previous work. These are:<br><br>0 - 20: Little or no burden<br>21 - 40: Light to moderate burden<br>41 - 60: Moderate to heavy burden<br>61 - 88: Heavy burden	
DEVELOPMENT<br>**Authors**<br>**Year**<br>**Country**	 Zarit [76]<br>1980<br>USA/ English	
LANGUAGES<br>**Translation/ Country**	☐ English<br>☒ German<br>☒ French<br>☒ Italian	[76]<br>[268, 269]<br>[348,349]<br>[274]
**Validation**	☒ German<br>☒ French<br>☒ Italian<br>☒ English	[268, 269]<br>[349,350]<br>[274]<br>[76]
**Short versions**	1 Question<br>4 Questions<br>7 Questions<br>12 Questions	[309]<br>[351]<br>[309]<br>[306]
FOR TARGET GROUP:<br>Family caregivers of persons with	Schizophrenia [272,298]<br>Alzheimer's dementia [288,352]<br>Palliative Care [270]<br>Children with Autism [289]<br>Chronically ill elderly [290]<br>Brain Injury [296]	
AVAILABILITY OF THE INSTRUMENT<br>**Paper Pencil and/ or digital version**	Freely accessible<br>☒ Paper Pencil, PDF for printing<br>☒ Digital, online questionnaire	

**Transferability**	Suitable for different target groups, also for parents of sick and impaired children.
UNDERLYING THEORETICAL MODELS	--
CONTACT	See authors of the studies
LITERATURE	

76. Zarit, S.H.; Reever, K.E.; Bach-Peterson, J. Relatives of the impaired elderly: Correlates of feelings of burden. Gerontologist 1980, 20, 649–655. https://doi.org/10.1093/geront/20.6.649.

268. Braun, M.; Scholz, U.; Hornung, R.; Martin, M. Caregiver burden with dementia patients. A validation study of the German language version of the Zarit Burden Interview. Zeitschrift für Gerontologie und Geriatrie 2010, 43, 111–119, doi:10.1007/s00391-010-0097-6.

269. Braun, M.; Scholz, U.; Hornung, R.; Martin, M. The burden of spousal caregiving: a preliminary psychometric evaluation of the German version of the Zarit burden interview. Aging & Mental Health 2010, 14, 159–167, doi:10.1080/13607860802459781.

270. Brink, P.; Stones, M.; Smith, T.F. Confirmatory factor analysis of the burden interview of the caregivers of terminally ill home care clients. Journal of palliative medicine 2012, 15, 967–970, doi:10.1089/jpm.2012.0086.

272. Caqueo-Urizar, A.; Gutierrez-Maldonado, J. Burden of care in families of patients with schizophrenia. Quality of Life Research 2006, 15, 719–724, doi:10.1007/s11136-005-4629-2.

274. Chattat, R.; Cortesi, V.; Izzicupo, F.; Del Re, M.L.; Sgarbi, C.; Fabbo, A.; Bergonzini, E. The Italian version of the Zarit Burden Interview: a validation study. International Psychogeriatrics 2011, 23, 797–805, doi:10.1017/s1041610210002218.

288. Epstein-Lubow, G.; Davis, J.D.; Miller, I.W.; Tremont, G. Persisting burden predicts depressive symptoms in dementia caregivers. Journal of Geriatric Psychiatry and Neurology 2008, 21, 198–203, doi:10.1177/0891988708320972.

289. Ezzat, O. Quality of Life and Subjective Burden on Family Caregiver of Children with Autism. American Journal of Nursing Science 2017, 6, 33, doi:10.11648/j.ajns.20170601.15.

290. Faison, K.J.; Faria, S.H.; Frank, D. Caregivers of chronically ill elderly: perceived burden. Journal of community health nursing 1999, 16, 243–253, doi:10.1207/s15327655jchn1604_4.

296. Frischknecht, E. Oscar Online-Coaching. Entwicklung und erste Evaluation eines Internet-basierten Unterstützungsprogramms für Angehörige von Menschen mit einer im Erwachsenenalter erworbenen Hirnverletzung. Universität Bern, 2011.

298. Gater, A.; Rofail, D.; Marshall, C.; Tolley, C.; Abetz-Webb, L.; Zarit, S.H.; Berardo, C.G. Assessing the Impact of Caring for a Person with Schizophrenia: Development of the Schizophrenia Caregiver Questionnaire. The Patient - Patient-Centered Outcomes Research 2015, 8, 507–520, doi:10.1007/s40271-015-0114-3.

306. Higginson, I.J.; Gao, W.; Jackson, D.; Murray, J.; Harding, R. Short-form Zarit Caregiver Burden Interviews were valid in advanced conditions. Journal of Clinical Epidemiology 2010, 63, 535–542, doi:10.1016/j.jclinepi.2009.06.014.

309. Kühnel, M.B.; Ramsenthaler, C.; Bausewein, C.; Fegg, M.; Hodiamont, F. Validation of two short versions of the Zarit Burden Interview in the palliative care setting: a questionnaire to assess the burden of informal caregivers. Supportive Care in Cancer 2020, doi:10.1007/s00520-019-05288-w.

349. Hébert, R.; Bravo, G.; Girouard, D. Fidélité de la traduction française de trois instruments d'évaluation des aidants naturels de malades déments. Canadian Journal on Aging / La Revue canadienne du vieil-lissement 1993, 12, 324–337, doi:10.1017/s0714980800013726.

350. Hébert, R.; Bravo, G.; Préville, M. Reliability, Validity and Reference Values of the Zarit Burden Interview for Assessing Informal Caregivers of Community-Dwelling Older Persons with Dementia. Canadian Journal on Aging / La Revue canadienne du vieillissement 2000, 19, 494–507, doi:10.1017/s0714980800012484.

351. Bédard, M.; Molloy, D.W.; Squire, L.; Dubois, S.; Lever, J.A.; O'Donnell, M. The Zarit Burden Interview: a new short version and screening version. The Gerontologist 2001, 41, 652–657, doi:10.1093/geront/41.5.652.

352. Campbell, P.; Wright, J.; Oyebode, J.; Job, D.; Crome, P.; Bentham, P.; Jones, L.; Lendon, C. Determinants of burden in those who care for someone with dementia. International journal of geriatric psychiatry 2008, 23, 1078–1085, doi:10.1002/gps.2071.
